# In-Frame cDNA Library Combined with Protein Complementation Assay Identifies ARL11-Binding Partners

**DOI:** 10.1371/journal.pone.0052290

**Published:** 2012-12-18

**Authors:** Sangkyou Lee, Ilkyun Lee, Yoonsuh Jung, David McConkey, Bogdan Czerniak

**Affiliations:** 1 Department of Pathology, The University of Texas MD Anderson Cancer Center, Houston, Texas, United States of America; 2 Department of Biostatistics, The University of Texas MD Anderson Cancer Center, Houston, Texas, United States of America; 3 Department of Urology, The University of Texas MD Anderson Cancer Center, Houston, Texas, United States of America; Colorado State University, United States of America

## Abstract

The cDNA expression libraries that produce correct proteins are essential in facilitating the identification of protein-protein interactions. The 5′-untranslated regions (UTRs) that are present in the majority of mammalian and non-mammalian genes are predicted to alter the expression of correct proteins from cDNA libraries. We developed a novel cDNA expression library from which 5′-UTRs were removed using a mixture of polymerase chain reaction primers that complement the Kozak sequences we refer to as an “in-frame cDNA library.” We used this library with the protein complementation assay to identify two novel binding partners for ras-related ADP-ribosylation factor-like 11 (ARL11), cellular retinoic acid binding protein 2 (CRABP2), and phosphoglycerate mutase 1 (PGAM1). Thus, the in-frame cDNA library without 5′-UTRs we describe here increases the chance of correctly identifying protein interactions and will have wide applications in both mammalian and non-mammalian detection systems.

## Introduction

**Figure 1:**
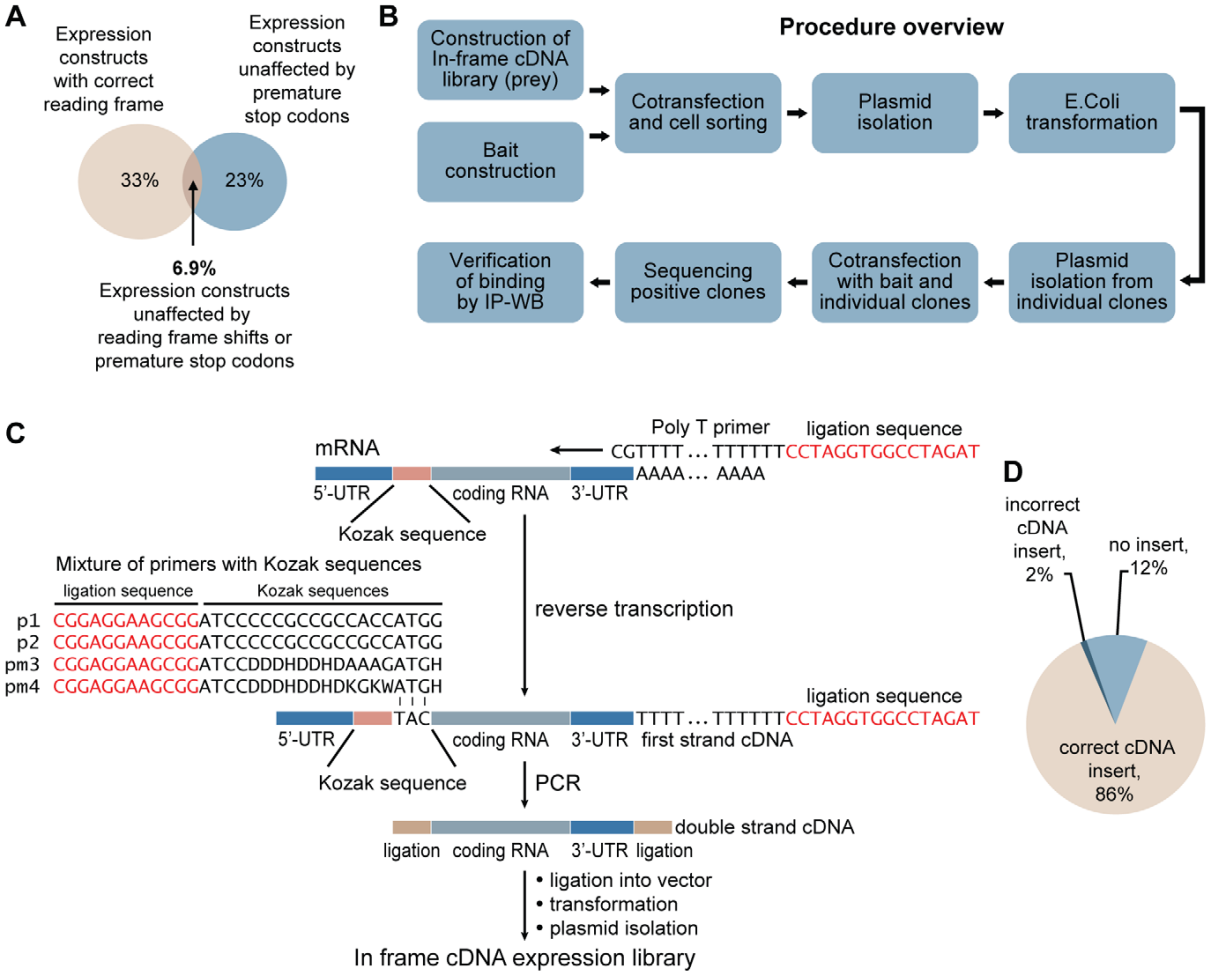
Analysis of the human 5′-UTR database, overview of the approach, and construction of the in-frame cDNA expression library. (**A**) Analysis of the human 5′-UTR database (http://utrdb.ba.itb.cnr.it/) to predict their effects on expressed sequences following translation with a YFP1 tag peptide as fusion proteins during the construction of a prey cDNA library. (**B**) Overview of the screening procedure. (**C**) For the construction of the in-frame cDNA expression library, mRNA was isolated from normal human urothelial cells and was used as a template for first-strand cDNA synthesis using polyT primer. Double-stranded cDNAs without 5′-UTRs were synthesized using primers 1 and 2 (representing approximately 40% of the Kozak sequences that are present in vertebrate genomes) complemented with primer mixes 3 and 4 (representing the remaining 60% of the Kozak sequence combinations in vertebrates). In primer mixes 3 and 4, the combination of sequences referred to as “D” is an equal mixture of A, G and T, “H” is an equal mixture of A, C and T, “K” is an equal mixture of G and T, and “W” is an equal mixture of A and T. There are 19,683 and 157,464 possible sequence combinations in primer mixes 3 and 4, respectively. (**D**) Sequence analysis of the in-frame cDNA library was performed on 198 representative plasmids isolated from random colonies of the library.

Identification of protein-protein interactions is an important issue of molecular biology in that it facilitates the studies of the function of these interactions in physiology and disease. In recognition of this fact, ambitious efforts were recently initiated to define the entire interactome [1]. The two main technologies employed – the yeast two-hybrid system [2] and the protein fragment complementation assay [3] – both utilize cDNA expression libraries. Therefore, the quality of the data obtained from these assays depends on the sequence fidelity of the polypeptides that are expressed from these cDNA libraries. Unfortunately, no attention has been paid to the possibility that the presence of 5′-untranslated region (UTR) sequences could affect the reading frames for the encoded protein in the expression constructs. We performed statistical analyses of the human 5′-UTR database which revealed that, when translated with a tag peptide as prey fusion proteins, a predicted 67% of constructs would be affected by a frame shift and 77% would contain premature stop codons. When we combined these analyses, less than 7% of expressed constructs were predicted to produce the correct full-length proteins **(** **Fig. 1A** **and Materials and Methods)**. The presence of sequence-altered proteins in these libraries almost certainly results in the identification of false protein–protein interactions and could prevent the identification of any interactions at all. Therefore, we consider the presence of 5′-UTRs within expressed gene open reading frames to be a major cause of both false-positive and false-negative results in technologies that utilize the bait-and-prey system of identifying interacting proteins [4].

Here we report on the design of a polymerase chain reaction (PCR)-based strategy to remove the 5′-UTR sequences from expression vectors by using a mixture of primers with Kozak sequences, which facilitates the construction of correct in-frame cDNA libraries [5]. We combined this approach with the protein complementation assay to identify novel protein-protein interactions **(** **Fig. 1B** **)**.

## Results and Discussion

Because our prior studies showed that downregulation of ras-related ADP-ribosylation factor-like 11 (ARL11) expression plays an important role in the early stages of human bladder carcinogenesis, we used RNA extracted from normal human urothelium to construct an in-frame cDNA library [6], [7]. First-strand cDNA was synthesized using a polyT primer, and double-stranded cDNAs without the 5′-UTRs were synthesized with the mixture of primers containing 177,149 possible combinations of the Kozak sequences present in vertebrate genomes [8] **(** **Fig. 1C** **and Materials and Methods)**. Sequence analyses of plasmids from the in-frame cDNA library performed on 198 plasmids isolated from random colonies documented the successful removal of 5′-UTRs from all inserts. Only 2% of cDNA inserts (4 inserts) had incorrect start codons **(** **Fig. 1D** **and Table S1)**. The two most frequent Kozak sequences (CCCGCCGCCACC**ATG**G; CCCGCCGCCGCC**ATG**G) in the human genome were identified in 49% and 37% of the sequenced expression constructs. **(Table S2)**.

In order to identify the proteins that interact with ARL11, we used yellow fluorescent protein fragment 1 (YFP1) as an N-terminal tag for proteins expressed by the in-frame library and ARL11 fused to yellow fluorescent protein fragment 2 (YFP2) at ARL11’s C-terminus as bait. We transiently co-transfected HEK-293T cells with the prey cDNA library and ARL11-YFP2. After 24 hours, we harvested the transfected cells and used flow cytometry to collect fluorescent cells. We collected only the brightest cells (approximately 1% of the total) for plasmid isolation. Before performing sequencing and partner protein identification, we transiently co-transfected the HEK-293T cells again with plasmids from individual colonies and ARL11-YFP2 to confirm the bait and prey interactions by fluorescence. We identified 27 plasmids encoding candidate ARL11-binding partners, which we subjected to amplification using DH5α competent *Escherichia coli* followed by final DNA sequencing to identify interacting proteins **(Tables S3 and S4)**. The data we obtained revealed sequences corresponding to five ribosomal binding proteins, most of which represented short fragments of coding sequences. In addition, there were three clones containing the full-length sequence of cellular retinoic acid binding protein 2 (*CRABP2*), a retinoic acid (RA) carrier protein that facilitates RA transfer from the cytosol to the nucleus and binding to its receptors [9], [10].

**Figure 2:**
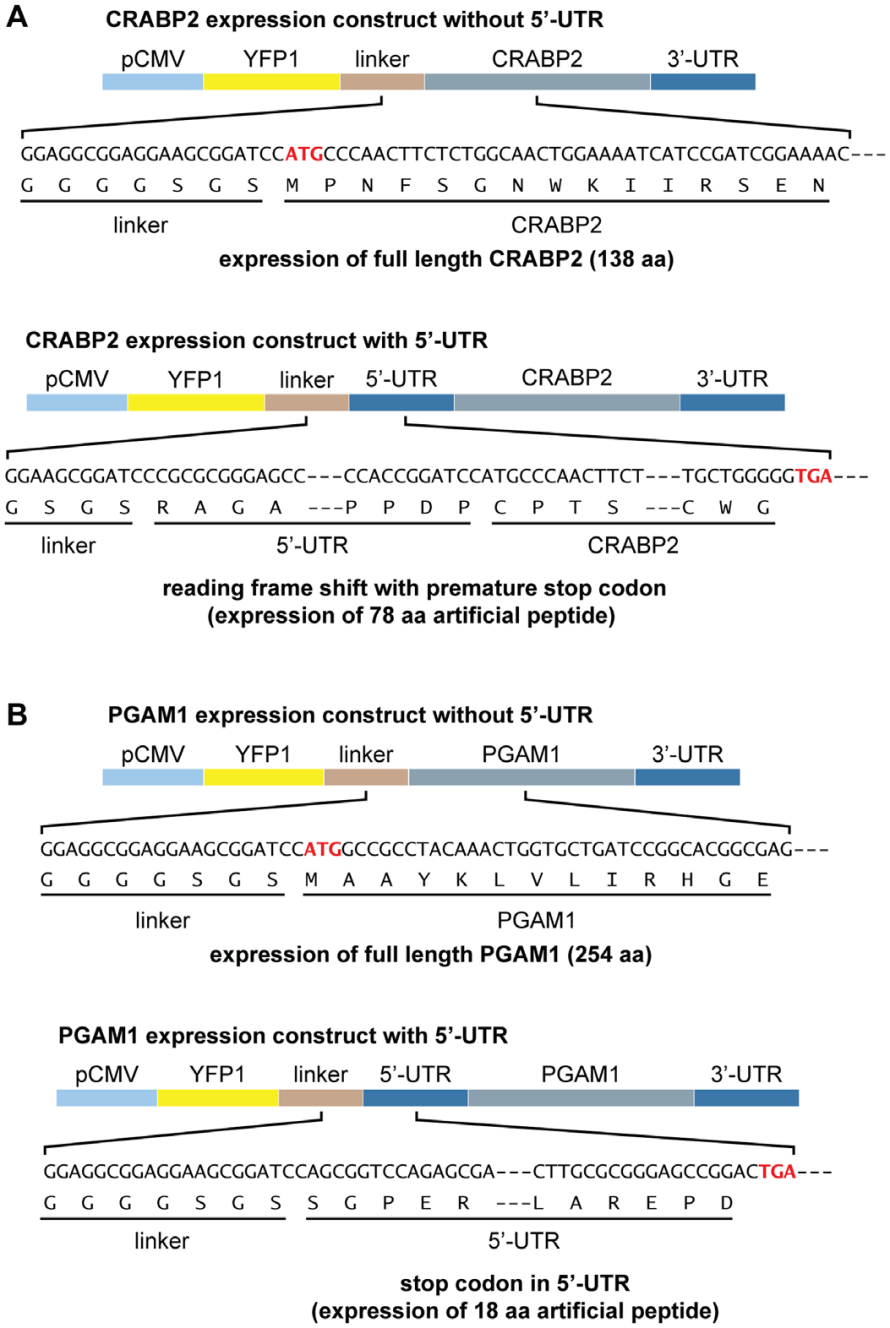
Predicted expression of CRABP2 and PGAM1 proteins by the constructs with and without 5′-UTRs. (**A**) Comparison of *CRABP2* expression constructs with and without the *CRABP2* 5′-UTR. (**B**) Comparison of *PGAM1* expression constructs with and without the *PGAM1* 5′-UTR.

**Figure 3:**
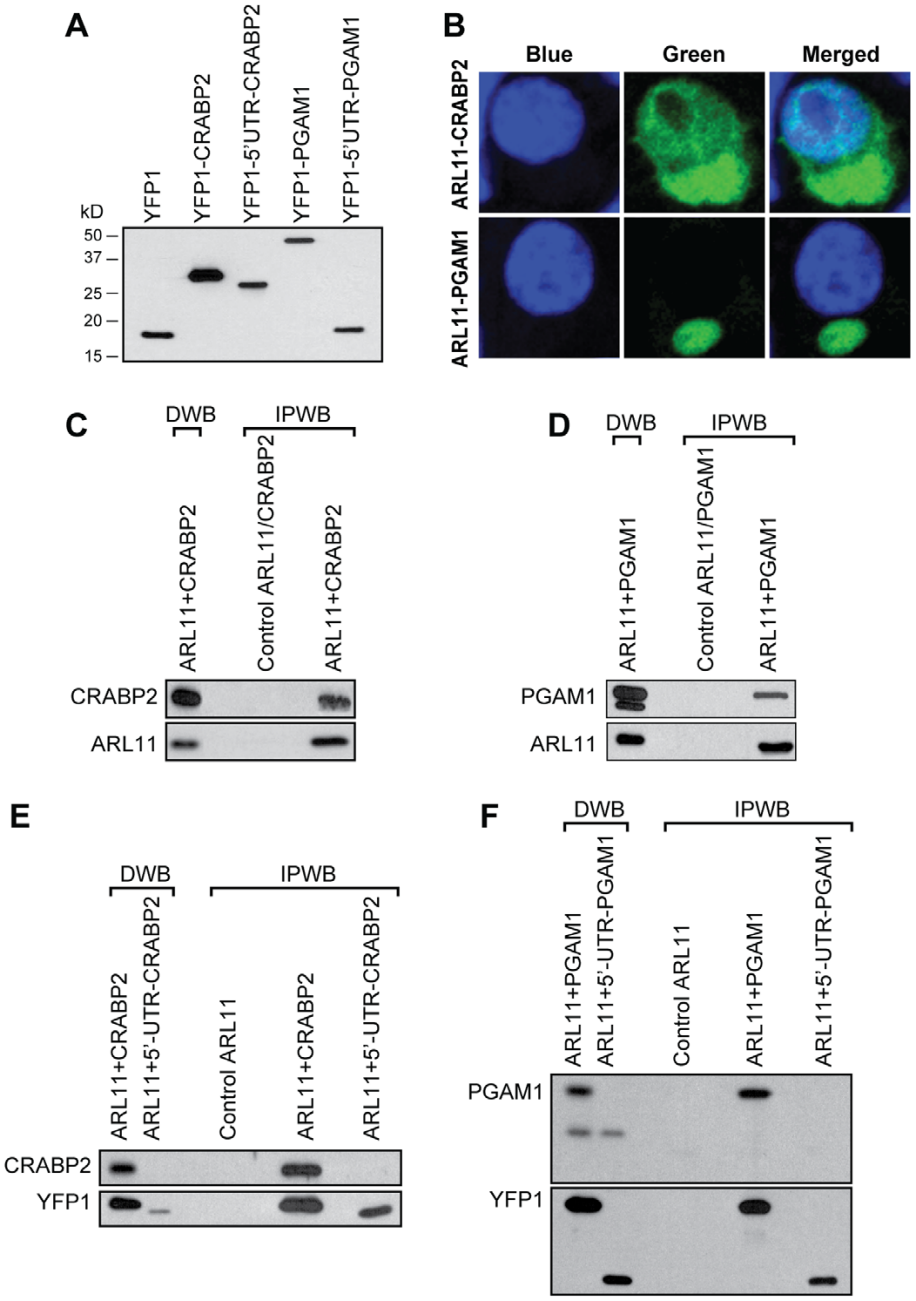
Identification of the CRABP2 and PGAM1 proteins as ARL11-binding partners using the in-frame cDNA expression library. (**A**) Western blot analysis of N-terminal YFP1-tagged fusion proteins expressed by the constructs with and without 5′-UTRs in HEK-293T cells. Expression of a construct containing only YFP1 was used as a control. An anti-GFP N-terminal antibody was used to visualize the expressed tagged proteins. (**B**) YFP fluorescence in HEK-293T cells after the co-transfection of YFP1-*CRABP2* with *ARL11*-YFP2 and YFP1-*PGAM1* with *ARL11*-YFP2. Nuclei were counterstained with DAPI. (**C**) Confirmation of the interaction between ARL11 and CRABP2 by western blotting and co-immunoprecipitation. HEK-293T cells were transfected with HA-tagged *ARL11* and FLAG-tagged *CRABP2* constructs without its 5′-UTR. Protein expression was verified by immunoblotting using anti-ARL11 and anti-CRABP2 antibodies in direct western blots (DWB). Immunoprecipitation with western blotting (IPWB) was performed by anti-HA antibody pull-down of ARL11 to detect CRABP2 binding (top panel). Results were confirmed using a complementary approach (HA-tagged CRABP2, anti-HA antibody immunoprecipitation, and anti-ARL11 immunoblotting (bottom panel). (**D**) Confirmation of ARL 11 and PGAM1 binding by IPWB. HEK-293T cells were transfected with HA-tagged *ARL11* and flag-tagged *PGAM1* constructs as indicated. Protein expression verified by immunoblotting with anti-PGAM1 (top panel) or anti-ARL11 (bottom panel) antibodies (DWBs). IPWBs were performed by anti-HA immunoprecipiation of ARL11 followed by immunoblotting with anti-PGAM1 (Top panel). Alternatively, immunoprecipitation was performed using HA-tagged PGAM1 followed by immunoblotting with anti-ARL11 (Bottom panel). (**E**) The in-frame cDNA library prevented interference caused by the *CRABP2* 5′-UTR that inhibits its binding to ARL11. HEK-293T cells were transfected with HA-*ARL11* and with YFP1-*CRABP2* or YFP1-5′-UTR-*CRABP2* as indicated. Protein expression was confirmed by immunoblotting with anti-CRABP2 antibody (top panel) or anti-YFP1 antibody (bottom panel). Alternatively, ARL11 was immunoprecipitated using the anti-HA antibody, and bound proteins were detected by immunoblotting with anti-CRABP2 (top panel) or anti-YFP1 (bottom panel) antibody. (**F**) The in-frame cDNA library prevents interference caused by the *PGAM1* 5′-UTR that prevents its binding to ARL11. HEK-293T cells were transfected with HA-*ARL11* and YFP1-*PGAM1*, or YFP1-5′-UTR-*PGAM1*. Protein expression was confirmed using anti-PGAM1 (top panel) or anti-YFP1 (bottom panel) antibodies (DWB). To identify ARL11-associated proteins (IPWB), ARL11 was immunoprecipitated using the anti-HA antibody and bound proteins were detected using either an anti-PGM1 (top panel) or anti-YFP (bottom panel) antibody.

A role for ARL11 in facilitating this transfer is consistent with the functions of other members of the ARF/ARL superfamily, which interact with intracellular membranes and facilitate protein and vesicular trafficking [11]. The idea that downregulation of ARL11 expression might promote early carcinogenesis via disruption of RA signaling is consistent with a very large body of work demonstrating that exogenous retinoids have chemopreventative effects in preclinical models and in patients with bladder cancer [12], [13], [14], [15], [16], [17]. The remaining clone contained the full-length sequence encoding phosphoglycerate mutase 1 (PGAM1), which catalyzes the reversible actions of 3-phosphoglycerate to 2-phosphoglycerate in the glycolytic pathway [18], [19]. The potential biological significance of its binding to ARL11 is unknown. However, because phosphorylation of PGAM1 is considered to play a role in the activation of an alternative glycolytic pathway of rapidly proliferating cells and phosphorylated PGAM1 has been shown to be overexpressed in these cells, phosphorylated PGAM1 represents a potential novel therapeutic target in several solid human tumors [20], [21], [22], [23], [24]. Finally, we examined whether the removal of 5′-UTRs from the constructs of *CRABP2* and *PGAM1* facilitated the identification of their interactions with ARL11. The presence of a 5′-UTR in the *CRABP2* insert construct caused a frame shift with a premature stop codon resulting in the expression of a 78-amino-acid artificial peptide **(** **Fig. 2A** **)**. For *PGAM1*, the presence of a 5′-UTR generated a stop codon within the 5′-UTR sequence and resulted in the expression of an 18-amino-acid artificial peptide **(** **Fig. 2B** **)**. Western blot analysis confirmed that the expression constructs for *CRABP2* and *PGAM1* with removed 5′-UTRs encoded full-length proteins while the constructs with 5′-UTRs caused the expression of smaller artificial peptides **(** **Fig. 3A** **and Materials and Methods)**.

Co-transfection of YFP1-*CRABP2* with YFP2-*ARL11* and YFP1-*PGAM1* with YFP2- *ARL11* fusion proteins into HEK-293T cells produced strong fluorescent signals confirming the interactions between these proteins **(** **Fig. 3B** **and Materials and Methods)**. CRABP2 is a cytosolic protein that moves into the nucleus upon binding with RA [9]. Our immunoflouresence data indicated that ARL11 binding to CRABP2 is associated with the cytosol-to-nucleus movement, but it is uncertain whether it plays any role in the reconfiguration of the functional nuclear localization signal of the CRABP2*-*RA-ARL11 complex. For PGAM1, the strong cytoplasmic immunoflouresence signal was consistent with the known cytosolic localization of the protein [19]. We further confirmed the interactions between ARL11 with CRABP2 and PGAM1 by co-immunoprecipitation. Proteins expressed by the constructs with removed 5′-UTRs expressing the correct full-length proteins were co-immunoprecipitated with the ARL11 protein **(** **Fig. 3C and 3D** **, and Materials and Methods)**.

In order to assess the interference of 5′-UTRs with the screening process of the cDNA library, we performed additional co-immunoprecipitation experiments using the expression constructs containing YFP1-tagged *CRABP2* and *PGAM1* inserts with and without 5′-UTRs. The full-length proteins expressed by the 5′-UTR-deleted constructs could again be co-immunoprecipitated with ARL11, whereas the artificial proteins expressed by the constructs containing 5′-UTRs produced non-specific interactions with ARL11 **(** **Fig. 3E and 3F** **, and Materials and Methods)**. The correct binding proteins could not be identified in the immunoprecipitates. Therefore, as predicted from the sequence analyses, the use of a cDNA library produced from mRNAs that contain 5′-UTRs would have interfered with the identification of the correct partner proteins for ARL11.

Techniques that measure interactions between proteins interrogate two partner proteins, called the bait and the prey, coupled to two halves of the transcription factor [2] and the two halves of the fluorescent protein [25]. If the proteins make contact, they reconstitute a transcriptional factor that activates a reporter gene in the yeast two-hybrid system or they reconstitute a flourescently active protein in the protein complementation assay. These two frequently used binary systems that measure interactions between a limited number of two proteins have recently been complemented by high-throughput platforms that can measure multiple binary interactions or interactions among groups of proteins [26], [27]. These platforms typically utilize a combination of luminescence or fluorescence tags with immunoprecipitation and mass spectrometry [26], [28], [29]. False-positive and false-negative interactions can skew the results, which is the main challenge in this field.

Initial efforts to address this issue were focused on improving the specificity of interactions with the transcriptional activator. In order to accomplish this, a new reporter gene, *CYC1-lacZ,* which contains three consensus binding sides for GAL4, was developed to reduce false-positive activations of the reporter gene [30]. In addition, a combination of co-transformations and yeast genetic mating techniques with several rounds of screenings was developed to confirm whether protein-protein interactions were correct [31]. The SOS recruitment system, which is based on the rescue of ras-mediated signal transduction in a cdc25-2 temperature-sensitive yeast strain, facilitates interactions between partner proteins in the cytoplasm as opposed to in the nucleus with the conventional yeast two-hybrid system [32]. Another approach to increase the specificity of protein interactions is based on the concept of a multiple reporter gene system utilizing a triple reporter assay with *URA3*, *MEL1* and *LacZ* genes [33]. A more recently developed approach to reduce false-positive reactions is based on the concept of permutated fusion proteins, which contain a mixture of N- and C-terminal bait fusion proteins [34]. Finally, because the cells used in the detection systems can activate the transcription of reporter genes by their internal proteins, the method of interrogating protein-protein interactions within the Golgi center, where internal transcriptional activation is reduced to a negligible background level, has been utilized [35]. All these approaches were designed to improve the specificity of transcriptional activation as well as the specificity of bait-and-prey interactions. They did not, however, address the problems related to the cDNA library construction with 5′-UTR-based frame shifts that may affect a large proportion of the expressed proteins. The in-frame cDNA library we have described here enables the effective removal of 5′-UTRs from the constructs, thereby facilitating the expression of correct proteins. The PCR approach utilizing primers with Kozak sequences permitted the capture of all the genes in the library, increasing the chance of identifying correct protein interactions. This system is anticipated to have wide applications in the detection of protein interactions utilizing genome-wide expression libraries across mammalian and non-mammalian species.

## Materials and Methods

### Cell Line and Human Tissue

All human tissues were collected with written informed consent under protocols approved by The University of Texas MD Anderson Cancer Center Institutional Review Board for this study, and the samples were analyzed anonymously. The HEK**-**293T cell line was obtained from the American Type Culture Collection and was maintained in Dulbecco’s modified Eagle’s medium supplemented with 10% fetal bovine serum in a humidified incubator under an atmosphere of 5% CO_2_ in air. Under an approved institutional protocol, normal human urothelial cells were obtained from ureters attached to nephrectomy specimens performed for excision of renal cell carcinomas without any evidence of the tumor involvement of the renal calyxes, pelvis, or ureter. The urothelial cell suspensions were prepared by scraping the urothelial surface and resuspending the cells in phosphate-buffered saline (PBS) as previously described [7].

### Analysis of Human 5′-UTR Database

Normally UTRs incorporated into the mRNA sequence do not cause shifts or premature stops in the reading frame. However, during the construction of a cDNA expression library, both 5′- and 3′-UTRs are incorporated into the sequences of the expression constructs, with the sequences encoding tag peptides, and can cause frame shifts or premature stop codons. Since the tag peptides and linkers were attached to the N-termini of the encoded proteins, we statistically analyzed the human 5′-UTR database in order to assess what proportion of the expressed proteins might be affected by the presence of 5′-UTRs fused with the tag and linker peptides in the typical cDNA expression library.

A 5′-UTR database was downloaded from http://utrdb.ba.itb.cnr.it/, which contained 124,102 variants of the 5′-UTR sequences identified in the human genome and was analyzed by the statistical software program R. For the analysis, T, C, G, and A bases were replaced by the numbers 1, 2, 3, and 4, respectively, and were converted into triplet codons. The proportion of genes affected by the presence of 5′-UTRs was calculated by dividing the number of bases in the 5′-UTRs by 3 and collecting those 5′-UTRs which had non-zero remainders. In order to calculate the number of 5′-UTRs which contained stop codons, we combined the bases into triplets as described above (starting from the first base) and identified the 5′-UTRs containing at least one stop codon.

### In-frame cDNA Expression Library

For the construction of an in-frame cDNA expression library, mRNA was isolated from human normal ureters using TRIzol reagent. (Life Technologies) The first-strand cDNA was synthesized using the polyT primer.

5′-TAGATCCGGTGGATCCTTTTTTTTTTTTTTTTTTTTTTTTTTTTTTGC-3′ and SMARTScribe reverse transcriptase (BD Bioscience Clontech). The polyT primer contained the TAGATCCGGTGGATC ligation sequence to facilitate the subsequent homologous recombination of the 3′ site of the amplified cDNAs. To synthesize double-stranded cDNAs without the 5′-UTRs, we used a mixture of forward primers reflecting the combination of Kozak sequences in vertebrate genomes [8] **(** **Table 1** **)**.

**Table 1:** Sequences of forward primers used to construct in-frame cDNA library.

Sequence Position	ligation sequence																	Kozak sequence*_____																
	5′-	1	2	3	4	5	6	7	8	9	10	11	12	13	14	15	16	17	18	19	20	21	22	23	24	25	26	27	28	29	30	31	32	3′
primer 1		C	G	G	A	G	G	A	A	G	C	G	G	A	T	C	C	C	C	C	G	C	C	G	C	C	A	C	C	A	T	G	G	
primer 2		C	G	G	A	G	G	A	A	G	C	G	G	A	T	C	C	C	C	C	G	C	C	G	C	C	G	C	C	A	T	G	G	
primer mix 3		C	G	G	A	G	G	A	A	G	C	G	G	A	T	C	C	**D**	**D**	**D**	**H**	**D**	**D**	**H**	**D**	A	A	A	G	A	T	G	**H**	
primer mix 4		C	G	G	A	G	G	A	A	G	C	G	G	A	T	C	C	**D**	**D**	**D**	**H**	**D**	**D**	**H**	**D**	**K**	G	**K**	**W**	A	T	G	**H**	
combination 1		C	G	G	A	G	G	A	A	G	C	G	G	A	T	C	C	**A**	A	A	C	A	A	C	A	G	G	G	A	A	T	G	C	
combination 2		C	G	G	A	G	G	A	A	G	C	G	G	A	T	C	C	**G**	A	A	C	A	A	C	A	G	G	G	A	A	T	G	C	
combination 3		C	G	G	A	G	G	A	A	G	C	G	G	A	T	C	C	**T**	A	A	C	A	A	C	A	G	G	G	A	A	T	G	C	
combination 4		C	G	G	A	G	G	A	A	G	C	G	G	A	T	C	C	**A**	**G**	A	C	A	A	C	A	G	G	G	A	A	T	G	C	
combination 5		C	G	G	A	G	G	A	A	G	C	G	G	A	T	C	C	**A**	**T**	A	C	A	A	C	A	G	G	G	A	A	T	G	C	
combination 6		C	G	G	A	G	G	A	A	G	C	G	G	A	T	C	C	**G**	**G**	A	C	A	A	C	A	G	G	G	A	A	T	G	C	
combination 7		C	G	G	A	G	G	A	A	G	C	G	G	A	T	C	C	**G**	**T**	A	C	A	A	C	A	G	G	G	A	A	T	G	C	
combination 8		C	G	G	A	G	G	A	A	G	C	G	G	A	T	C	C	**T**	**G**	A	C	A	A	C	A	G	G	G	A	A	T	G	C	
combination 9		C	G	G	A	G	G	A	A	G	C	G	G	A	T	C	C	**T**	**T**	A	C	A	A	C	A	G	G	G	A	A	T	G	C	

* D is an equal mixture of A, G and T, H is an equal mixture of A, C and T, K is an equal mixture of G and T, and W is an equal mixture of A and T.

All forward primers contained the same ligation sequence corresponding to positions 1–16, which facilitated the homologous recombination of the 5′ site of the amplified cDNAs. Positions 17–32 in the forward primers corresponded to sequence variations around the translational start site (positions −12 to +4) of the reported Kozak sequences [8]. The most frequent consensus sequences for the initiation of translation are in primers 1 and 2, which correspond to approximately 40% of Kozak sequences in vertebrates. In order to complement mRNAs containing the remaining combinations of the Kozak sequences, we designed the primer mixes 3 and 4 as sequence combinations reflecting all reported variations at positions 17–25, 27, 28, and 32 of the primer design. At position 17, the sequence frequency was reported as A 23%, G 23%, C 35%, and T 19%. Thus, an equal mixture of A, G, and T at position 17 was used in primer mixes 3 and 4 to approximate this frequency. The nucleotide C was excluded from the combinatorial design at position 17 because it was already included in the design of primers 1 and 2. Then, in a similar manner, the sequence combinations for positions 18–24 and 32 were designed to reflect their approximate relative frequencies in vertebrate genomes. At position 25, the frequency of A was 25%, G 15%, and T 7%. Because the frequency of A was much higher than G or T at this position, we selected nucleotide A at position 25 for primer mix 3 and the mixture of G and T at position 25 for primer mix 4. Similar combinations were designed for positions 27 and 28 in primer mixes 3 and 4.

In summary, in primer mixes 3 and 4, “D” was an equal mixture of A, G and T, “H” was an equal mixture of A, C and T, “K” was an equal mixture of G and T, and “W” was an equal mixture of A and T. There were 19,683 and 157,464 possible sequence combinations for primer mixes 3 and 4, respectively. We show nine sequence combinations for nucleotides at positions 17 and 18 in primer mix 4 as an example. Sequence combinations 1–3 at position 17 represent A, G, and T, respectively. All possible sequence combinations at position 17 are shown as combinations 4–9, and the sequence combinations with an equal frequency of A, G, and T are shown for position 18.

For PCR, primers 1 and 2 and primer mixes 3 and 4 were combined in the ratio of 24∶16:30∶30 to reflect their approximate relative proportions in vertebrate genomes. The PCR reaction mixture contained the forward primers, the PolyT reverse primer, and LA Taq DNA polymerase (BD Bioscience Clontech). The double-stranded cDNAs were amplified for 16 cycles using the following thermal profile: 94°C for 15 seconds, 42°C for 30 seconds, 72°C for 6 minutes.

The amplified double-stranded cDNA fragments were cloned into the pEGFP-cl vector (BD Bioscience Clontech) together with fragment 1 (YFP1 amino acids 1–158) of a yellow fluorescent protein variant (Addgene) and a 10-amino-acid linker consisting of (Gly-Gly-Gly-Gly-Ser)2x [36]. First, the *EGFP* cDNA was replaced with the Kozak sequence by using *Nhe*I and *Xho*I enzymes. The PCR-amplified *YFP1* cDNA was digested with *Xho*I and *Eco*RI to make cohesive 5′- and 3′ ends. The linker oligo DNA was synthesized to contain the cleaved *Eco*RI and *Bam*HI overhangs at the 5′- and 3′ ends. The *YFP1* cDNA with the linker DNA was cloned at the *Xho*I and *Bam*HI sites. Amplified double-stranded cDNAs from a ureter were then cloned at the *Bam*HI site using the In-Fusion cloning kit (BD Bioscience Clontech) to encode C-terminal fusion protein to YFP1. Subsequent bacterial transformation and amplification of the library was performed according to the protocols of the In-Fusion SMARTer cDNA library construction kit (BD Bioscience Clontech).

### Bait Construct

The pcDNA3.1 vector (Life Technologies) was used to construct the bait by inserting *ARL11* cDNA linked to the 10-amino-acid flexible linker consisting of (Gly-Gly-Gly-Gly-Ser)2x at *Nhe*I and *Xho*I sites, and YFP2 (amino acids 159–239) was inserted at the *Xho*I site to encode the C-terminal fusion YFP2.

### Screening of the In-frame cDNA Library

HEK-293T cells were transiently co-transfected with in-frame library and *ARL11*-YFP2 plasmids using Lipofectamine 2000 reagent (Life Technologies). After 24 hours, the transfected cells were harvested, washed with PBS, and sorted to collect fluorescent cells using a FACSAria cell sorter (BD Biosciences). Only the brightest (top 1%) fluorescent cells were collected, and plasmids were recovered from the sorted cells. DH5α competent *E. coli* were transformed with the plasmids, and the individual colonies were picked for plasmid isolation. Subsequently, HEK-293T cells were transiently co-transfected with the isolated plasmids and the *ARL11*-YFP2 plasmid to confirm the identified protein-protein interactions. Only those plasmids which showed strong positive fluorescence were submitted for sequencing and identification of the binding partners.

### ARL11-binding Protein Expression

The cDNAs of the candidate ARL11-binding proteins identified in the screen (*CRABP2* and *PGAM1*) were used to prepare expression constructs that were then employed to validate their interactions with ARL11. pEGFP-c1 expression constructs with and without 5′-UTRs containing N-terminal YFP1 were prepared to assess the potential interference of the 5′-UTRs with the expression of the correct proteins and their interaction with ARL11 by using fluorescent assays. In addition, to verify the interactions between CRABP2 and PGAM1 with ARL11 by immunoprecipitation, hemagglutinin (HA)- and FLAG-tagged plasmids of *ARL11, CRABP2,* and *PGAM1* with and without 5′-UTRs were prepared. HA- or FLAG-tagged DNA with *ARL11* cDNA was cloned into the EGFP-c1 vector after removal of the *EGFP* cDNA. All remaining constructs were made by replacing the YFP1 tag with an HA or FLAG tag.

### Fluorescence Experiments

To verify the binding between ARL11 and partner proteins, HEK-293T cells growing on glass cover slips were transiently co-transfected with plasmids containing YFP1-*CRABP2*, YFP1-5′-UTR-*CRABP2*, YFP1-*PGAM1*, or YFP1-5′-UTR-*PGAM1* and *ARL11*-YFP2. After 24 hours, the cells were fixed in 4% paraformaldehyde for 10 minutes, washed with PBS, stained with DAPI, and analyzed with a Leica TCS SP5 confocal microscope (Leica Microsystems).

### Immunoprecipitation

For co-immunoprecipitation of ARL11 and its binding partner proteins, HEK-293T cells were transiently co-transfected with plasmids encoding HA-*ARL11* and either FLAG-*CRABP2* or FLAG-*PGAM1*. Transfection with plasmids expressing HA-*ARL11* served as the control. After 24 hours, cells were lysed by sonication in an immunoprecipitation buffer (137 mM NaCl, 10 mM phosphate, 2.7 mM KCl, and protease inhibitors). Anti-HA antibody agarose beads (Sigma-Aldrich, E6779; 30 µl) were added to the lysate (800 µg of total protein) to immunoprecipitate ARL11. After incubation at 4°C for 5 hours, the beads were washed thrice with the immunoprecipitation buffer and boiled with Laemmli’s reducing sample buffer for 4 minutes. Protein samples were separated by SDS-PAGE and transferred to nitrocellulose membranes. The membranes were probed with either anti-CRABP2 antibody (BD Pharmingen, 560234; 1∶3,000) or anti-PGAM1 antibody (Sigma-Aldrich, Sab1100295; 1∶2,500).

To confirm the specificities of the interactions, reciprocal immunoprecipitation experiments were performed. Specifically, HEK-293T cells were transiently co-transfected with FLAG-tagged ARL11 and HA-tagged CRABP2 or PGAM1. Transfection with plasmids encoding HA-tagged CRABP2 or PGAM1 served as controls. Lysates were immunoprecipitated using anti-HA antibody agarose beads as described above, and ARL11 was detected using an anti-ARL11 antibody developed in our laboratory.

To address whether the presence of 5′-UTRs in the expression constructs altered interactions between partner proteins, HEK-293T cells were transiently co-transfected with plasmids encoding HA-*ARL11* and YFP1-*CRABP2*, YFP1-5′-UTR-*CRABP2*, YFP1-*PGAM1*, or YFP1-5′-UTR-*PGAM1*. Transfections with only the plasmids encoding HA-*ARL11* served as control. Immunoprecipitation was performed using the anti-HA antibody agarose beads as described above, and the membranes were probed with anti-CRABP2 (Sigma-Aldrich, C6873∶1:2,000), anti-PGAM1 (Sigma-Aldrich, Sab1100295; 1∶2,500), or anti-GFP N-terminal (Sigma-Aldrich, G1544; 1∶1,000) antibody.

## Supporting Information

Table S1 **Kozak sequences from position 17 to 32 plus first 3 codons of 174 random in-frame cDNA library clones.**
(DOC)

Table S2 **Kozak sequence analysis of 174 random in-frame cDNA library clones.**
(DOC)

Table S3 **DNA sequences of clones identified as putative ARL11 binders.**
(DOC)

Table S4 **Clones identified as putative ARL11 binders.**
(DOC)
