# Supplementary material for: In-Frame cDNA Library Combined with Protein Complementation Assay Identifies ARL11-Binding Partners
Source: PLoS One. 2012 Dec 18;7(12):e52290. doi: 10.1371/journal.pone.0052290 (PMC3525598; doi:10.1371/journal.pone.0052290)
Supplement: Table S1 — Kozak sequences from position 17 to 32 plus first 3 codons of 174 random in-frame cDNA library clones. (DOC) [file pone.0052290.s001.doc]

Table S1. Kozak sequences from position  17 to 32 plus first 3 codons of 174 random in-frame cDNA library clones
Gene name	No. of clones	Number	Ref Sequence #	Kozak sequence plus first 3 codons*	
Ribosomal protein S12 (RPS12)	18				
		1	NM_001016.3	CCCGCCGCCGCCATG GCC GAG GAA	
		2	NM_001016.3	CCCGCCGCCACCATG GCC GAG GAA	
		3	NM_001016.3	CCCGCCGCCACCATG GCC GAG GAA	
		4	NM_001016.3	CCCGCCGCCACCATG GCC GAG GAA	
		5	NM_001016.3	CCCGCCGCCGCCATG GCC GAG GAA	
		6	NM_001016.3	CCCGCCGCCACCATG GCC GAG GAA	
		7	NM_001016.3	CCCGCCGCCACCATG GCC GAG GAA	
		8	NM_001016.3	CCCGCCGCCGCCATG GCC GAG GAA	
		9	NM_001016.3	CCCGCCGCCACCATG GCC GAG GAA	
		10	NM_001016.3	CCCGCCGCCACCATG GCC GAG GAA	
		11	NM_001016.3	CCCGCCGCCGCCATG GCC GAG GAA	
		12	NM_001016.3	CCCGCCGCCACCATG GCC GAG GAA	
		13	NM_001016.3	CCCGCCGCCACCATG GCC GAG GAA	
		14	NM_001016.3	CCCGCCGCCACCATG GCC GAG GAA	
		15	NM_001016.3	CCCGCCGCCACCATG GCC GAG GAA	
		16	NM_001016.3	CCCGCCGCCACCATG GCC GAG GAA	
		17	NM_001016.3	CCCGCCGCCGCCATG GCC GAG GAA	
		18	NM_001016.3	CCCGCCGCCGCCATG GCC GAG GAA	
Guanine nucleotide binding protein (G protein), beta polypeptide 2-like 1 (GNB2L1)	12				
		19	NM_006098.4	CCCGCCGCCGCCATG GCT GAG CAG	
		20	NM_006098.4	CCCGCCGCCACCATG GCT GAG CAG	
		21	NM_006098.4	CCCGCCGCCGCCATG GCT GAG CAG	
		22	NM_006098.4	CCCGCCGCCACCATG GCT GAG CAG	
		23	NM_006098.4	CCCGCCGCCACCATG GCT GAG CAG	
		24	NM_006098.4	CCCGCCGCCGCCATG ACT GAG CAG	
		25	NM_006098.4	CCCGCCGCCACCATG GCT GAG CAG	
		26	NM_006098.4	CCCGCCGCCACCATG GCT GAG CAG	
		27	NM_006098.4	CCCGCCGCCGCCATG GCT GAG CAG	
		28	NM_006098.4	CCCGCCGCCACCATG GCT GAG CAG	
		29	NM_006098.4	CCCGCCGCCGCCATG GCT GAG CAG	
		30	NM_006098.4	CCCGCCGCCGCCATG ACT GAG CAG	
Chloride intracellular channel 1 (CLIC1)	9				
		31	NM_001288.4	CCCGCCGCCGCCATG GCT GAA GAA	
		32	NM_001288.4	CCCGCCGCCACCATG GCT GAA GAA	
		33	NM_001288.4	CCCGCCGCCACCATG GCT GAA GAA	
		34	NM_001288.4	CCCGCCGCCGCCATG GCT GAA GAA	
		35	NM_001288.4	CCCGCCGCCGCCATG GCT GAA GAA	
		36	NM_001288.4	CCCGCCGCCGCCATG GCT GAA GAA	
		37	NM_001288.4	CCCGCCGCCACCATG GCT GAA GAA	
		38	NM_001288.4	CCCGCCGCCGCCATG GCT GAA GAA	
		39	NM_001288.4	CCCGCCGCCGCCATG GCT GAA GAA	
Ribosomal protein L14 (RPL14), transcript variant 2	8				
		40	NM_003973.3	CCCGCCGCCACCATG GTG TTC AGG	
		41	NM_003973.3	CCCGCCGCCACCATG GTG TTC AGG	
		42	NM_003973.3	CCCGCCGCCGCCATG GTG TTC AGG	
		43	NM_003973.3	CCCGCCGCCACCATG GTG TTC AGG	
		44	NM_003973.3	CCCGCCGCCACCATG GTG TTC AGG	
		45	NM_003973.3	CCCGCCGCCGCCATG GTG TTC AGG	
		46	NM_003973.3	CCCGCCGCCACCATG GTG TTC AGG	
		47	NM_003973.3	CCCGCCGCCGCCATG GTG TTC AGG	
Eukaryotic translation elongation factor 2 (EEF2)	7				
		48	NM_001961.3	CCCGCCGCCACCATG GTG AAC TTC	
		49	NM_001961.3	CCCGCCGCCGCCATG GTG AAC TTC	
		50	NM_001961.3	CCCGCCGCCACCATG GTG AAC TTC	
		51	NM_001961.3	CCCGCCGCCACCATG GTG AAC TTC	
		52	NM_001961.3	CCCGCCGCCACCATG GTG AAC TTC	
		53	NM_001961.3	CCCGCCGCCGCCATG GTG AAC TTC	
		54	NM_001961.3	CCCGCCGCCGCCATG GTG AAC TTC	
Ribosomal protein S28 (RPS28)	6				
		55	NM_001031.4	CCCGCCGCCACCATG GAC ACC AGC	
		56	NM_001031.4	CCCGCCGCCGCCATG GAC ACC AGC	
		57	NM_001031.4	CCCGCCGCCGCCATG GTC TTC AGG	
		58	NM_001031.4	CCCGCCGCCACCATG GAC ACC AGC	
		59	NM_001031.4	CCCGCCGCCGCCATG GAC ACC AGC	
		60	NM_001031.4	CCCGCCGCCACCATG GAC ACC AGC	
Cellular retinoic acid binding protein 2 (CRABP2), transcript variant 1	5				
		61	NM_001878.3	CCCGCCGCCACCATG CCC AAC TTC	
		62	NM_001878.3	CCCGCCGCCGCCATG GCC AAC TTC	
		63	NM_001878.3	CCCGCCGCCGCCATG CCC AAC TTC	
		64	NM_001878.3	CCCGCCGCCGCCATG GCC AAC TTC	
		65	NM_001878.3	CCCGCCGCCACCATG GCC AAC TTC	
Actin, beta (ACTB)	4				
		66	NM_001101.3	CCCGCCGCCACCATG GAT GAT GAT	
		67	NM_001101.3	CCCGCCGCCACCATG GAT GAT GAT	
		68	NM_001101.3	CCCGCCGCCACCATG GAT GAT GAT	
		69	NM_001101.3	CCCGCCGCCGCCATG GAT GAT GAT	


Mitochondrion	4				
		70	NC_012920.1	CCCGCCGCCGCCATG GCC TCC ATG	
		71	NC_012920.1	CCCGCCGCCACCATG GCT TTT TCA	
		72	NC_012920.1	CCCGCCGCCACCATG GCC TCC ATG	
		73	NC_012920.1	CCCGCCGCCGCCATG GCC TCC ATG	
Ribosomal protein L28 (RPL28), transcript variant 2	4				
		74	NM_000991.4	CCCGCCGCCACCATG TCT GCG CAT	
		75	NM_000991.4	CCCGCCGCCACCATG TCT GCG CAT	
		76	NM_000991.4	CCCGCCGCCACCATG TCT GCG CAT	
		77	NM_000991.4	CCCGCCGCCGCCATG TCT GCG CAT	
Transgelin 2 (TAGLN2)	4				
		78	NM_003564.1	CCCGCCGCCGCCATG GCC TTC AAG	
		79	NM_003564.1	CCCGCCGCCGCCATG GCC TTC AAG	
		80	NM_003564.1	CCCGCCGCCACCATG GCC TTC AGG	
		81	NM_003564.1	CCCGCCGCCACCATG GCC TTC AAG	
Activating transcription factor 4 (tax-responsive enhancer element B67) (ATF4), transcript variant 2	3				
		82	NM_182810.1	CCCGCCGCCGCCATG GCG TAT TAG	
		83	NM_182810.1	CCCGCCGCCGCCATG GCG TAT TAG	
		84	NM_182810.1	CCCGCCGCCACCATG GCG TAT TAG	
FtsJ homolog 3 (E. coli) (FTSJ3)	3				
		85	NM_017647.3	CCCGCCGCCGCCATG GGC AAG AAG	
		86	NM_017647.3	CCCGCCGCCGCCATG GGC AAG AAG	
		87	NM_017647.3	CCCGCCGCCACCATG GGC AAG AAG	
Phosphoglycerate mutase 1 (brain) (PGAM1)	3				
		88	NM_002629.2	CCCGCCGCCGCCATG GCC GCC TAC	
		89	NM_002629.2	CCCGCCGCCGCCATG GCC GCC TAC	
		90	NM_002629.2	CCCGCCGCCACCATG GCC GCC TAC	
Guanosine monophosphate reductase 2 (GMPR2), transcript variant 1	3				
		91	NM_016576.3	CCCGCCGCCGCCATG GCA GGG AAT	
		92	NM_016576.3	CCCGCCGCCACCATG GAT GCT GTA	
		93	NM_016576.3	CCCGCCGCCACCATG GCA GGG AAT	
Glutathione S-transferase pi 1 (GSTP1)	2				
		94	NM_000852.3	CCCGCCGCCGCCATG CCG CCC TAC	
		95	NM_000852.3	CCCGCCGCCACCATG GCG CCC TAC	


Ribosomal protein L22 (RPL22)	2				
		96	NM_000983.3	CCCGCCGCCACCATG GCT CCT GTG	
		97	NM_000983.3	CCCGCCGCCGCCATG GCT CCT GTG	
Ribosomal protein L14 (RPL14), transcript variant 1	2				
		98	NM_001034996.1	CCCGCCGCCACCATG GTG AGT CTT	
		99	NM_001034996.1	CCCGCCGCCGCCATG GTG AGT CTT	
Ribosomal protein L28 (RPL28), transcript variant 1	2				
		100	NM_001136134.1	CCCGCCGCCGCCATG GGC TCC TGA	
		101	NM_001136134.1	CCCGCCGCCACCATG GGC TCC TGA	
Phosphogluconate dehydrogenase (PGD)	2				
		102	NM_002631.2	CCCGCCGCCGCCATG GCC CAA GCT	
		103	NM_002631.2	CCCGCCGCCACCATG GCC CAA GCT	
RAB13, member RAS oncogene family (RAB13)	2				
		104	NM_002870.2	CCCGCCGCCACCATG GCC AAA GCC	
		105	NM_002870.2	CCCGCCGCCGCCATG GCC AAA GCC	
CD81 molecule (CD81)	2				
		106	NM_004356.3	CCCGCCGCCACCATG GGA GTG GAG	
		107	NM_004356.3	CCCGCCGCCACCATG GGA GTG GAG	
Tubulin, beta 2C (TUBB2C)	2				
		108	NM_006088.5	GCCGCCGCCATCATG AGG GAA ATC	
		109	NM_006088.5	CCCGCCGCCGCCATG GTG AGG GAA	
Guanine nucleotide binding protein (G protein), alpha inhibiting activity polypeptide 3 (GNAI3)	2				
		110	NM_006496.2	CCCGCCGCCACCATG GGC TGC ACG	
		111	NM_006496.2	CCCGCCGCCACCATG GGC TGC ACG	
Ring finger protein 7 (RNF7), transcript variant 1	2				
		112	NM_014245.4	CCCGCCGCCGCCATG GCC GAC GTG	
		113	NM_014245.4	CCCGCCGCCGCCATG GCC GAC GTG	
PTEN induced putative kinase 1 (PINK1), nuclear gene encoding mitochondrial protein	2				
		114	NM_032409.2	CCCGCCGCCGCCATG GTG CTG CTG	
		115	NM_032409.2	CCCGCCGCCGCCATG GTG CTG CTG	


Hypothetical LOC100270710 (LOC100270710), non-coding rRNA	2				
		116	NR_026754.1	CCCGCCGCCACCATG GAG CCT CAT	
		117	NR_026754.1	CCCGCCGCCACCATG GAG CCT CAT	
Chromosome 1 genomic contig	2				
		118	NT_004610.19	CCCGCCGCCACCATG GCT TCC ATC	
		119	NT_004610.19	CCCGCCGCCACCATG GTG TTT AAT	
Chromosome 2 genomic  contig	2				
		120	NT_005334.16	CCCGCCGCCACCATG GAC CAA GCA	
		121	NT_005334.16	AGGATATTAAAGATG CGG CAT TCC	
Acetyl-CoA acyltransferase 1 (ACAA1), nuclear gene encoding mitochondrial protein, transcript variant 1	1	122	NM_001607.3	CCCGCCGCCGCCATG GAG GGC CTG	
Aminopeptidase puromycin sensitive (NPEPPS)	1	123	NM_006310.3	GGTTGGTGAAAGATG TCT TTT CAC	
Arachidonate 15-lipoxygenase (ALOX15)	1	124	NM_001140.3	CCCGCCGCCGCCATG GAT GCA ACG	
Bromodomain PHD finger transcription factor (BPTF), transcript variant 1	1	125	NM_182641.3	CCCGCCGCCACCATG GAA GAA AGA	
Cold inducible RNA binding protein (CIRBP), transcript variant1 	1	126	NM_001280.2	CCCGCCGCCACCATG GCA TCA GAT	
Cytochrome b, ascorbate dependent 3 (CYBASC3), transcript variant 3	1	127	NM_001161452.1	CCCGCCGCCACCATG GCT GCT TGC	
Eukaryotic translation elongation factor 1 beta 2 (EEF1B2), transcript variant 1	1	128	NM_001959.3	CCCGCCGCCACCATG GGT TTC GGA	
Family with sequence similarity 199, X-linked (FAM199X)	1	129	NM_207318.3	CCCGCCGCCGCCATG GCA TTA ACC	
FK506 binding protein 1A, 12kDa (FKBP1A), transcript variant 1	1	130	NM_000801.4	CCCGCCGCCACCATG GGA GTG CAG	
GABA(A) receptor-associated protein-like 2 (GABARAPL2)	1	131	NM_007285.6	CCCGCCGCCACCATG GAG TGG ATG	
Heat shock protein 90kDa alpha (cytosolic), class B member 1 (HSP90AB1)	1	132	NM_007355.2	CCCGCCGCCACCTGT CTG AGC TGC	


High mobility group nucleosomal binding domain 2 (HMGN2)	1	133	NM_005517.3	CCCGCCGCCACCATG GCC AAG AGA	
Keratin 17 (KRT17)	1	134	NM_000422.2	CCCGCCGCCGCCATG GCC ACC TCC	
Keratin 18 (KRT18)	1	135	NM_000224.2	CCCGCCGCCGCCATG GTC CGC AAA	
Keratin 19 (KRT19)	1	136	NM_002276.4	ATGATTATAAAGATG CCT TCC GAA	
Mediator of cell motility 1 (MEMO1), transcript variant 1	1	137	NM_015955.2	CCCGCCGCCACCATC TCC TCA TTC	
Mitogen-activated protein kinase 3 (MAPK3), transcript variant 2	1	138	NM_001040056.1	CCCGCCGCCACCATG GAG CTG GAT	
Poly(rC) binding protein 1 (PCBP1)	1	139	NM_006196.3	CCCGCCGCCGCCATG GAT GCC GGT	
Polymerase (RNA) II (DNA directed) polypeptide L, 7.6kDa (POLR2L)	1	140	NM_021128.4	CCCGCCGCCACCATG GTC ATC CCT	
Protein arginine methyltransferase 5 (PRMT5), transcript variant 1	1	141	NM_006109.3	AATTGTAGAAAGATG CGG CGA TGG	
Pyridoxal-dependent decarboxylase domain containing 2, pseudogene (PDXDC2P), non-coding RNA	1	142	NR_003610.1	GGGTGGAGAAAGATG TCC CAG TGC	
Ribosomal protein L8 (RPL8), transcript variant 2	1	143	NM_033301.1	GTGTGGTTAAAGATG TGG ACG GAG	
RAB1B, member RAS oncogene family (RAB1B)	1	144	NM_030981.2	CCCGCCGCCACCATG GAC CCC GAA	
Ribosomal protein S13 (RPS13)	1	145	NM_001017.2	CCCGCCGCCACCATG GGT CGA AGG	
Ribosomal protein S18 (RPS18)	1	146	NM_022551.2	CCCGCCGCCACCTGT GCT GCA GCC	
Ribosomal protein, large, P1 (RPLP1), transcript variant 1	1	147	NM_001003.2	CCCGCCGCCGCCATG GCC TCT GTC	
Signal transducer and activator of transcription 3 (acute-phase response factor) (STAT3), transcript variant 3	1	148	NM_213662.1	CCCGCCGCCACCATG GCT GGC TAG	
Slingshot homolog 3 (Drosophila) (SSH3)	1	149	NM_017857.3	CCCGCCGCCACCATG GCT TCC TGC	
Small nuclear ribonucleoprotein 200kDa (U5) (SNRNP200)	1	150	NM_014014.4	CCCGCCGCCGCCATG GCC AAG CCT	
Transketolase (TKT), transcript variant 1	1	151	NM_001064.3	CCCGCCGCCACCATG GAG AGC TAC	


Ubiquinol-cytochrome c reductase, complex III subunit VII, 9.5kDa (UQCRQ), nuclear gene encoding mitochondrial protein	1	152	NM_014402.4	CCCGCCGCCGCCATG GGC CGC GAG	
Zinc finger, AN1-type domain 3 (ZFAND3)	1	153	NM_021943.2	GGTTTATGGGGAATG AGG CCC CTG	
Chromosome 12 genomic contig	1	154	NT_029419.12	CCCGCCGCCACCATG GCC TAC CTG	
Chromosome 13 genomic contig	1	155	NW_001838074.1	CCCGCCGCCACCATG GAG CCA AGC	
Chromosome 15 genomic contig	1	156	NW_001838219.1	CCCGCCGCCACCATG GCC AAC AGG	
Chromosome 18 genomic contig	1	157	NT_010966.14	CCCGCCGCCACCATG GTT GAA CTA	
Chromosome 2 genomic contig	1	158	NT_005403.17	AGTCGGTGAAAGATG TAG CAT TTC	
Chromosome 20 genomic contig	1	159	NW_001838664.2	CCCGCCGCCACCATG GCT GTC ACA	
Chromosome 22 open reading frame 28 (C22orf28)	1	160	NM_014306.4	CCCGCCGCCGCCATG GGA ACC TGT	
Chromosome 3 genomic contig	1	161	NT_022459.15	CCCGCCGCCGCCATG GAG ACA GGA	
Chromosome 6 genomic contig	1	162	NT_025741.15	CCCGCCGCCGCCATG GCC GAG GAA	
Chromosome 6 genomic contig	1	163	NW_001838973.1	CCCGCCGCCACCATG GAC CAC CCC	
Chromosome 9 genomic contig	1	164	NW_001839237.2	CCCGCCGCCACCATG GCA TCT TCA	
Chromosome 9 open reading frame 142 (C9orf142)	1	165	NM_183241.1	CCCGCCGCCACCATC ACC TGC CTG	
Chromosome X genomic contig	1	166	NT_167197.1	GGATGGTGAAAGATG TAG ACG GGG	
No homology	1	167		CCCGCCGCCACCATG GCG AAA CCC	
Not available	1	168	Not available	CCCGCCGCCACCATG GCC AAG GAA	
Not available	1	169	Not available	CCCGCCGCCGCCATG GAC TAC GAA	
Not available	1	170	Not available	CCCGCCGCCGCCATG GTG TTC ACG	
Not available	1	171	Not available	CCCGCCGCCGCCATG GCC TCC ATG	
Not available	1	172	Not available	CCCGCCGCCACCATG GCC TCC ATG	
Not available	1	173	Not available	CCCGCCGCCACCATG GCC TCC ATG	
Not available	1	174	Not available	CCCGCCGCCGCCATG GCC GCC TAC	
*Start codon is underlined.
