# Supplementary material for: In-Frame cDNA Library Combined with Protein Complementation Assay Identifies ARL11-Binding Partners
Source: PLoS One. 2012 Dec 18;7(12):e52290. doi: 10.1371/journal.pone.0052290 (PMC3525598; doi:10.1371/journal.pone.0052290)
Supplement: Table S2 — Kozak sequence analysis of 174 random in-frame cDNA library clones. (DOC) [file pone.0052290.s002.doc]

**Table S2. Kozak sequence analysis of 174** **random in-frame cDNA library clones**

| **Number** | **Kozak sequence (base position from 17 to 32)*** | **Number of clones, description** |
| --- | --- | --- |
| 1 | CCCGCCGCCACC**ATG**G | 86 |
| 2 | CCCGCCGCCGCC**ATG**G | 65 |
| 3 | CCCGCCGCCACC**ATG**T | 3 |
| 4 | CCCGCCGCCGCC**ATG**A | 2 |
| 5 | CCCGCCGCCGCC**ATG**C | 2 |
| 6 | AATTGTAGAAAG**ATG**C | 1 |
| 7 | AGGATATTAAAG**ATG**C | 1 |
| 8 | AGTCGGTGAAAG**ATG**T | 1 |
| 9 | ATGATTATAAAG**ATG**C | 1 |
| 10 | CCCGCCGCCACC**ATG**C | 1 |
| 11 | CCCGCCGCCGCC**ATG**T | 1 |
| 12 | GCCGCCGCCATC**ATG**A | 1 |
| 13 | GGATGGTGAAAG**ATG**T | 1 |
| 14 | GGGTGGAGAAAG**ATG**T | 1 |
| 15 | GGTTGGTGAAAG**ATG**T | 1 |
| 16 | GGTTTATGGGGA**ATG**A | 1 |
| 17 | GTGTGGTTAAAG**ATG**T | 1 |
| 18 | CCCGCCGCCACC**ATC**A | 1, start codon error |
| 19 | CCCGCCGCCACC**ATC**T | 1, start codon error |
| 20 | CCCGCCGCCACC**TGT**C | 1 , start codon error |
| 21 | CCCGCCGCCACC**TGT**G | 1 , start codon error |

*Start codon is underlined.
