# Supplementary material for: In-Frame cDNA Library Combined with Protein Complementation Assay Identifies ARL11-Binding Partners
Source: PLoS One. 2012 Dec 18;7(12):e52290. doi: 10.1371/journal.pone.0052290 (PMC3525598; doi:10.1371/journal.pone.0052290)
Supplement: Table S3 — DNA sequences of clones identified as putative ARL11 binders. (DOC) [file pone.0052290.s003.doc]

**Table S3. DNA sequences of clones identified as putative ARL11 binders**

1. Cellular retinoic acid binding protein 2

Query 1 ATGCCCAACTTCTCTGGCAACTGGAAAATCATCCGATCGGAAAACTTCGAGGAATTGCTC 60

||||||||||||||||||||||||||||||||||||||||||||||||||||||||||||

Sbjct 222 ATGCCCAACTTCTCTGGCAACTGGAAAATCATCCGATCGGAAAACTTCGAGGAATTGCTC 281

Query 61 AAAGTGCTGGGGGTGAATGTGATGCTGAGGAAGATTGCTGTGGCTGCAGCGTCCAAGCCA 120

||||||||||||||||||||||||||||||||||||||||||||||||||||||||||||

Sbjct 282 AAAGTGCTGGGGGTGAATGTGATGCTGAGGAAGATTGCTGTGGCTGCAGCGTCCAAGCCA 341

Query 121 GCAGTGGAGATCAAACAGGAGGGAGACACTTTCTACATCAAAACCTCCACCACCGTGCGC 180

||||||||||||||||||||||||||||||||||||||||||||||||||||||||||||

Sbjct 342 GCAGTGGAGATCAAACAGGAGGGAGACACTTTCTACATCAAAACCTCCACCACCGTGCGC 401

Query 181 ACCACAGAGATTAACTTCAAGGTTGGGGAGGAGTTTGAGGAGCAGACTGTGGATGGGAGG 240

||||||||||||||||||||||||||||||||||||||||||||||||||||||||||||

Sbjct 402 ACCACAGAGATTAACTTCAAGGTTGGGGAGGAGTTTGAGGAGCAGACTGTGGATGGGAGG 461

Query 241 CCCTGTAAGAGCCTGGTGAAATGGGAGAGTGAGAATAAAATGGTCTGTGAGCAGAAGCTC 300

||||||||||||||||||||||||||||||||||||||||||||||||||||||||||||

Sbjct 462 CCCTGTAAGAGCCTGGTGAAATGGGAGAGTGAGAATAAAATGGTCTGTGAGCAGAAGCTC 521

Query 301 CTGAAGGGAGAGGGCCCCAAGACCTCGTGGACCAGAGAACTGACCAACGATGGGGAACTG 360

||||||||||||||||||||||||||||||||||||||||||||||||||||||||||||

Sbjct 522 CTGAAGGGAGAGGGCCCCAAGACCTCGTGGACCAGAGAACTGACCAACGATGGGGAACTG 581

Query 361 ATCCTGACCATGACGGCGGATGACGTTGTGTGCACCAGGGTCTACGTCCGAGAGTGAGTG 420

||||||||||||||||||||||||||||||||||||||||||||||||||||||||||||

Sbjct 582 ATCCTGACCATGACGGCGGATGACGTTGTGTGCACCAGGGTCTACGTCCGAGAGTGAGTG 641

Query 421 GCCACAGGTAGAACCGCGGCCGAAGCCCACCACTGGCCATGCTCACCGCCCTGCTTCACT 480

||||||||||||||||||||||||||||||||||||||||||||||||||||||||||||

Sbjct 642 GCCACAGGTAGAACCGCGGCCGAAGCCCACCACTGGCCATGCTCACCGCCCTGCTTCACT 701

Query 481 GCCCCCTCCGTCCCACCCCCTCCTTCTAGGATAGCGCTCCCCTTACCCCAGTCACTTCTG 540

||||||||||||||||||||||||||||||||||||||||||||||||||||||||||||

Sbjct 702 GCCCCCTCCGTCCCACCCCCTCCTTCTAGGATAGCGCTCCCCTTACCCCAGTCACTTCTG 761

Query 541 GGGGTCACTGGGATGCCTCTTGCAGGGTCTTGCTTTCTTTGACCTCTTCTCTCCTCCCCT 600

||||||||||||||||||||||||||||||||||||||||||||||||||||||||||||

Sbjct 762 GGGGTCACTGGGATGCCTCTTGCAGGGTCTTGCTTTCTTTGACCTCTTCTCTCCTCCCCT 821

Query 601 ACACCAACAAAGAGGAATGGCTGCAAGAGCCCAGATCACCCATTCCGGGTTCACTCCCCG 660

||||||||||||||||||||||||||||||||||||||||||||||||||||||||||||

Sbjct 822 ACACCAACAAAGAGGAATGGCTGCAAGAGCCCAGATCACCCATTCCGGGTTCACTCCCCG 881

Query 661 CCTCCCCAAGTCAGCAGTCCTAGCCCCAAACCAGCCCAGAGCAGGGTCTCTCTAAAGGGG 720

||||||||||||||||||||||||||||||||||||||||||||||||||||||||||||

Sbjct 882 CCTCCCCAAGTCAGCAGTCCTAGCCCCAAACCAGCCCAGAGCAGGGTCTCTCTAAAGGGG 941

Query 721 ACTTGAGGGCCTGAGCAGGAAAGACTGGCCCTCTAGCTTCTACCCTTTGTCCCTGTAGCC 780

||||||||||||||||||||||||||||||||||||||||||||||||||||||||||||

Sbjct 942 ACTTGAGGGCCTGAGCAGGAAAGACTGGCCCTCTAGCTTCTACCCTTTGTCCCTGTAGCC 1001

Query 781 TATACAGTTTAGAATATTTATTTGTTAATTTTATTAAAATGC 822

||||||||||||||||||||||||||||||||||||||||||

Sbjct 1002 TATACAGTTTAGAATATTTATTTGTTAATTTTATTAAAATGC 1043

1. Cellular retinoic acid binding protein 2

Query 1 ATGGCCAACTTCTCTGGCAACTGGAAAATCATCCGATCGGAAAACTTCGAGGGATTGCTC 60

||| |||||||||||||||||||||||||||||||||||||||||||||||| |||||||

Sbjct 222 ATGCCCAACTTCTCTGGCAACTGGAAAATCATCCGATCGGAAAACTTCGAGGAATTGCTC 281

Query 61 AAAGTGCTGGGGGTGAATGTGATGCTGAGGAAGATTGCTGCGGCTGCAGCGTCCAAGCCA 120

|||||||||||||||||||||||||||||||||||||||| |||||||||||||||||||

Sbjct 282 AAAGTGCTGGGGGTGAATGTGATGCTGAGGAAGATTGCTGTGGCTGCAGCGTCCAAGCCA 341

Query 121 GCAGTGGAGATCAAACAGGAGGGAGACACTTTCTACATCAAAACCTCCACCACCGTGCGC 180

||||||||||||||||||||||||||||||||||||||||||||||||||||||||||||

Sbjct 342 GCAGTGGAGATCAAACAGGAGGGAGACACTTTCTACATCAAAACCTCCACCACCGTGCGC 401

Query 181 ACCACAGAGATTAACTTCAAGGTTGGGGAGGAGTTTGAGGAGCAGACTGTGGATGGGAGG 240

||||||||||||||||||||||||||||||||||||||||||||||||||||||||||||

Sbjct 402 ACCACAGAGATTAACTTCAAGGTTGGGGAGGAGTTTGAGGAGCAGACTGTGGATGGGAGG 461

Query 241 CCCTGTAAGAGCCTGGTGAAATGGGAGAGTGAGAATAAAATGGTCTGTGAGCAGAAGCTC 300

||||||||||||||||||||||||||||||||||||||||||||||||||||||||||||

Sbjct 462 CCCTGTAAGAGCCTGGTGAAATGGGAGAGTGAGAATAAAATGGTCTGTGAGCAGAAGCTC 521

Query 301 CTGAAGGGAGAGGGCCCCAAGACCTCGTGGACCAGAGAACTGACCAACGATGGGGAACTG 360

||||||||||||||||||||||||||||||||||||||||||||||||||||||||||||

Sbjct 522 CTGAAGGGAGAGGGCCCCAAGACCTCGTGGACCAGAGAACTGACCAACGATGGGGAACTG 581

Query 361 ATCCTGACCATGACGGCGGATGACGTTGTGTGCACCAGGGTCTACGTCCGAGAGTGAGTG 420

||||||||||||||||||||||||||||||||||||||||||||||||||||||||||||

Sbjct 582 ATCCTGACCATGACGGCGGATGACGTTGTGTGCACCAGGGTCTACGTCCGAGAGTGAGTG 641

Query 421 GCCACAGGTAGAACCGCGGCCGAAGCCCACCACTGGCCATGCTCACCGCCCTGCTTCACT 480

||||||||||||||||||||||||||||||||||||||||||||||||||||||||||||

Sbjct 642 GCCACAGGTAGAACCGCGGCCGAAGCCCACCACTGGCCATGCTCACCGCCCTGCTTCACT 701

Query 481 GCCCCCTCCGTCCCACCCCCTCCTTCTAGGATAGCGCTCCCCTTACCCCAGTCACTTCTG 540

||||||||||||||||||||||||||||||||||||||||||||||||||||||||||||

Sbjct 702 GCCCCCTCCGTCCCACCCCCTCCTTCTAGGATAGCGCTCCCCTTACCCCAGTCACTTCTG 761

Query 541 GGGGTCACTGGGATGCCTCTTGCAGGGTCTTGCTTTCTTTGACCTCTTCTCTCCTCCCCT 600

||||||||||||||||||||||||||||||||||||||||||||||||||||||||||||

Sbjct 762 GGGGTCACTGGGATGCCTCTTGCAGGGTCTTGCTTTCTTTGACCTCTTCTCTCCTCCCCT 821

Query 601 ACACCAACAAAGAGGAATGGCTGCAAGAGCCCAGATCACCCATTCCGGGTTCACTCCCCG 660

||||||||||||||||||||||||||||||||||||||||||||||||||||||||||||

Sbjct 822 ACACCAACAAAGAGGAATGGCTGCAAGAGCCCAGATCACCCATTCCGGGTTCACTCCCCG 881

Query 661 CCTCCCCAAGTCAGCAGTCCTAGCCCCAAACCAGCCCAGAGCAGGGTCTCTCTAAAGGGG 720

||||||||||||||||||||||||||||||||||||||||||||||||||||||||||||

Sbjct 882 CCTCCCCAAGTCAGCAGTCCTAGCCCCAAACCAGCCCAGAGCAGGGTCTCTCTAAAGGGG 941

Query 721 ACTTGAGGGCCTGAGCAGGAAAGACTGGCCCTCTAGCTTCTACCCTTTGTCCCTGTAGCC 780

||||||||||||||||||||||||||||||||||||||||||||||||||||||||||||

Sbjct 942 ACTTGAGGGCCTGAGCAGGAAAGACTGGCCCTCTAGCTTCTACCCTTTGTCCCTGTAGCC 1001

Query 781 TATACAGTTTAGAATATTTATTTGTTAATTTTATTAAAATGC 822

||||||||||||||||||||||||||||||||||||||||||

Sbjct 1002 TATACAGTTTAGAATATTTATTTGTTAATTTTATTAAAATGC 1043

1. Cellular retinoic acid binding protein 2

Query 1 ATGGCCAACTTCTCTGGCAACTGGAAAATCATCCGATCGGAAAACTTCGAGGAATTGCTC 60

||| ||||||||||||||||||||||||||||||||||||||||||||||||||||||||

Sbjct 222 ATGCCCAACTTCTCTGGCAACTGGAAAATCATCCGATCGGAAAACTTCGAGGAATTGCTC 281

Query 61 AAAGTGCTGGGGGTGAATGTGATGCTGAGGAAGATTGCTGTGGCTGCAGCGTCCAAGCCA 120

||||||||||||||||||||||||||||||||||||||||||||||||||||||||||||

Sbjct 282 AAAGTGCTGGGGGTGAATGTGATGCTGAGGAAGATTGCTGTGGCTGCAGCGTCCAAGCCA 341

Query 121 GCAGTGGAGATCAAACAGGAGGGAGACACTTTCTACATCAAAACCTCCACCACCGTGCGC 180

||||||||||||||||||||||||||||||||||||||||||||||||||||||||||||

Sbjct 342 GCAGTGGAGATCAAACAGGAGGGAGACACTTTCTACATCAAAACCTCCACCACCGTGCGC 401

Query 181 ACCACAGAGATTAGCTTCAAGGTTGGGGAGGAGTTTGAGGAGCAGACTGTGGATGGGAGG 240

||||||||||||| ||||||||||||||||||||||||||||||||||||||||||||||

Sbjct 402 ACCACAGAGATTAACTTCAAGGTTGGGGAGGAGTTTGAGGAGCAGACTGTGGATGGGAGG 461

Query 241 CCCTGTAAGAGCCTGGTGAAATGGGAGAGTGAGAATAAAATGGTCTGTGAGCAGAAGCTC 300

||||||||||||||||||||||||||||||||||||||||||||||||||||||||||||

Sbjct 462 CCCTGTAAGAGCCTGGTGAAATGGGAGAGTGAGAATAAAATGGTCTGTGAGCAGAAGCTC 521

Query 301 CTGAAGGGAGAGGGCCCCAAGACCTCGTGGACCAGAGAACTGACCAACGATGGGGAACTG 360

||||||||||||||||||||||||||||||||||||||||||||||||||||||||||||

Sbjct 522 CTGAAGGGAGAGGGCCCCAAGACCTCGTGGACCAGAGAACTGACCAACGATGGGGAACTG 581

Query 361 ATCCTGACCATGACGGCGGATGACGTTGTGTGCACCAGGGTCTACGTCCGAGAGTGAGTG 420

||||||||||||||||||||||||||||||||||||||||||||||||||||||||||||

Sbjct 582 ATCCTGACCATGACGGCGGATGACGTTGTGTGCACCAGGGTCTACGTCCGAGAGTGAGTG 641

Query 421 GCCACAGGTAGAACCGCGGCCGAAGCCCACCACTGGCCATGCTCACCGCCCTGCTTCACT 480

||||||||||||||||||||||||||||||||||||||||||||||||||||||||||||

Sbjct 642 GCCACAGGTAGAACCGCGGCCGAAGCCCACCACTGGCCATGCTCACCGCCCTGCTTCACT 701

Query 481 GCCCCCTCCGCCCCACCCCCTCCTTCTAGGATAGCGCTCCCCTTACCCCAGTCACTTCTG 540

|||||||||| |||||||||||||||||||||||||||||||||||||||||||||||||

Sbjct 702 GCCCCCTCCGTCCCACCCCCTCCTTCTAGGATAGCGCTCCCCTTACCCCAGTCACTTCTG 761

Query 541 GGGGTCACTGGGATGCCTCTTGCAGGGTCTTGCTTTCTTTGACCTCTTCTCTCCTCCCCT 600

||||||||||||||||||||||||||||||||||||||||||||||||||||||||||||

Sbjct 762 GGGGTCACTGGGATGCCTCTTGCAGGGTCTTGCTTTCTTTGACCTCTTCTCTCCTCCCCT 821

Query 601 ACACCAACAAAGAGGAATGGCTGCAAGAGCCCAGATCACCCATTCCGGGTTCACTCCCCG 660

||||||||||||||||||||||||||||||||||||||||||||||||||||||||||||

Sbjct 822 ACACCAACAAAGAGGAATGGCTGCAAGAGCCCAGATCACCCATTCCGGGTTCACTCCCCG 881

Query 661 CCTCCCCAAGTCAGCAGTCCTAGCCCCAAACCAGCCCAGAGCAGGGTCTCTCTAAAGGGG 720

||||||||||||||||||||||||||||||||||||||||||||||||||||||||||||

Sbjct 882 CCTCCCCAAGTCAGCAGTCCTAGCCCCAAACCAGCCCAGAGCAGGGTCTCTCTAAAGGGG 941

Query 721 ACTTGAGGGCCTGAGCAGGAAAGACTGGCCCTCTAGCTTCTACCCTTTGTCCCTGTAGCC 780

||||||||||||||||||||||||||||||||||||||||||||||||||||||||||||

Sbjct 942 ACTTGAGGGCCTGAGCAGGAAAGACTGGCCCTCTAGCTTCTACCCTTTGTCCCTGTAGCC 1001

Query 781 TATACAGTTTAGAATATTTATTTGTTAATTTTATTAAAATGC 822

||||||||||||||||||||||||||||||||||||||||||

Sbjct 1002 TATACAGTTTAGAATATTTATTTGTTAATTTTATTAAAATGC 1043

1. Phosphoglycerate mutase 1

Query 1 ATGGCCGCCTACAAACTGGTGCTGATCCGGCACGGCGAGAGCGCATGGAACCTGGAGAAC 60

||||||||||||||||||||||||||||||||||||||||||||||||||||||||||||

Sbjct 39 ATGGCCGCCTACAAACTGGTGCTGATCCGGCACGGCGAGAGCGCATGGAACCTGGAGAAC 98

Query 61 CGCTTCAGCGGCTGGTACGACGCCGACCTGAGCCCGGCGGGCCACGAGGAGGCGAAGCGC 120

||||||||||||||||||||||||||||||||||||||||||||||||||||||||||||

Sbjct 99 CGCTTCAGCGGCTGGTACGACGCCGACCTGAGCCCGGCGGGCCACGAGGAGGCGAAGCGC 158

Query 121 GGCGGGCAGGCGCTACGAGATGCTGGCTATGAGTTTGACATCTGCTTCACCTCAGTGCAG 180

||||||||||||||||||||||||||||||||||||||||||||||||||||||||||||

Sbjct 159 GGCGGGCAGGCGCTACGAGATGCTGGCTATGAGTTTGACATCTGCTTCACCTCAGTGCAG 218

Query 181 AAGAGAGCGATCCGGACCCTCTGGACAGTGCTAGATGCCATTGATCAGATGTGGCTGCCA 240

||||||||||||||||||||||||||||||||||||||||||||||||||||||||||||

Sbjct 219 AAGAGAGCGATCCGGACCCTCTGGACAGTGCTAGATGCCATTGATCAGATGTGGCTGCCA 278

Query 241 GTGGTGAGGACTTGGCGCCTCAATGAGCGGCACTATGGGGG**G**TCTAACCGGTCTCAATAA 300

||||||||||||||||||||||||||||||||||||||||| ||||||||||||||||||

Sbjct 279 GTGGTGAGGACTTGGCGCCTCAATGAGCGGCACTATGGGGG**-**TCTAACCGGTCTCAATAA 337

Query 301 AGCAGAAACTGCTGCAAAGCATGGTGAGGCCCAGGTGAAGATCTGGAGGCGCTCCTATGA 360

||||||||||||||||||||||||||||||||||||||||||||||||||||||||||||

Sbjct 338 AGCAGAAACTGCTGCAAAGCATGGTGAGGCCCAGGTGAAGATCTGGAGGCGCTCCTATGA 397

Query 361 TGTCCCACCACCTCCGATGGAGCCCGACCATCCTTTCTACAGCAACATCAGTAAGGATCG 420

||||||||||||||||||||||||||||||||||||||||||||||||||||||||||||

Sbjct 398 TGTCCCACCACCTCCGATGGAGCCCGACCATCCTTTCTACAGCAACATCAGTAAGGATCG 457

1. Testis expressed 261

Query 2 TGGCATACTACACAGATCACTCTGGGCTCACTTGCCTGCCTAATGGTCATCTCCCCAGTA 61

||||||||||||||||||||||||||||||||||||||||||||||||||||||||||||

Sbjct 3196 TGGCATACTACACAGATCACTCTGGGCTCACTTGCCTGCCTAATGGTCATCTCCCCAGTA 3255

Query 62 GACTGTAAGCTCCTTGAGGGCAAGGATTGTGTTGGAATTTTTGTATTAACAGTGCCTGGC 121

||||||||||||||||||||||||||||||||||||||||||||||||||||||||||||

Sbjct 3256 GACTGTAAGCTCCTTGAGGGCAAGGATTGTGTTGGAATTTTTGTATTAACAGTGCCTGGC 3315

Query 122 TTGGTGCCTGGCACCTAGAAAGCACTCAATAAATGTTTGTTTAATG 167

||||||||||||||||||||||||||||||||||||||||||||||

Sbjct 3316 TTGGTGCCTGGCACCTAGAAAGCACTCAATAAATGTTTGTTTAATG 3361

1. Hypothetical LOC100507645, transcript variant 2

Query 1 ATGTATTTAAAAGAAAATTGAGAGAAAGGACTACAGAGCCCCGAATTAATACCAATAGAA 60

||||||||||||||||||||||||||||||||||||||||||||||||||||||||||||

Sbjct 1641 ATGTATTTAAAAGAAAATTGAGAGAAAGGACTACAGAGCCCCGAATTAATACCAATAGAA 1582

Query 61 GGGCAATGCTTTTAGATTAAAATGAAGGTGACTTAAACAGCTTAAAGTTTAGTTTAAAAG 120

||||||||||||||||||||||||||||||||||||||||||||||||||||||||||||

Sbjct 1581 GGGCAATGCTTTTAGATTAAAATGAAGGTGACTTAAACAGCTTAAAGTTTAGTTTAAAAG 1522

Query 121 TTGTAGGTGATTAAAATAATTTGAAGGCGATCTTTTAAAAAGAGATTAAACCGAAGGTGA 180

||||||||||||||||||||||||||||||||||||||||||||||||||||||||||||

Sbjct 1521 TTGTAGGTGATTAAAATAATTTGAAGGCGATCTTTTAAAAAGAGATTAAACCGAAGGTGA 1462

Query 181 TTAAAAGACCTTGAAATCCATGACGCAGGGAGAATTGCGTCATTTAAAGCCTAGTTAACG 240

||||||||||||||||||||||||||||||||||||||||||||||||||||||||||||

Sbjct 1461 TTAAAAGACCTTGAAATCCATGACGCAGGGAGAATTGCGTCATTTAAAGCCTAGTTAACG 1402

Query 241 CATTTACTAAACGCAGACGAAAATGGAAAGATTAATTGGGAGTGGTAGGATGAAACAATT 300

||||||||||||||||||||||||||||||||||||||||||||||||||||||||||||

Sbjct 1401 CATTTACTAAACGCAGACGAAAATGGAAAGATTAATTGGGAGTGGTAGGATGAAACAATT 1342

Query 301 TGGAGAAGATAGAAGTTTGAAGTGGAAAACTGGGAAGACAGAAGTACGGGAAGGC 355

||||||||||||||||||||||||||||||||| |||||||||||||||||||||

Sbjct 1341 TGGAGAAGATAGAAGTTTGAAGTGGAAAACTGG-AAGACAGAAGTACGGGAAGGC 1288

1. Ribosomal protein S11

Query 1 ATGCAGATGCAGAGGACCATTGTCATCCGCCGAGACTATCTGCACTACATCCGCAAGTAC 60

||| ||||||||||||||||||||||||||||||||||||||||||||||||||||||||

Sbjct 318 ATGAAGATGCAGAGGACCATTGTCATCCGCCGAGACTATCTGCACTACATCCGCAAGTAC 377

Query 61 AACCGCTTCGAGAAGCGCCACAAGAACATGTCTGTACACCTGTCCCCCTGCTTCAGGGAC 120

||||||||||||||||||||||||||||||||||||||||||||||||||||||||||||

Sbjct 378 AACCGCTTCGAGAAGCGCCACAAGAACATGTCTGTACACCTGTCCCCCTGCTTCAGGGAC 437

Query 121 GTCCAGATCGGTGACATCGTCACAGTGGGCGAGTGCCGGCCTCTGAGCAAGACAGTGCGC 180

||||||||||||||||||||||||||||||||||||||||||||||||||||||||||||

Sbjct 438 GTCCAGATCGGTGACATCGTCACAGTGGGCGAGTGCCGGCCTCTGAGCAAGACAGTGCGC 497

Query 181 TTCAACGTGCTCAAGGTCACCAAGGCTGCCGGCACCAAGAAGCAGTTCCAGAAGTTCTGA 240

||||||||||||||||||||||||||||||||||||||||||||||||||||||||||||

Sbjct 498 TTCAACGTGCTCAAGGTCACCAAGGCTGCCGGCACCAAGAAGCAGTTCCAGAAGTTCTGA 557

Query 241 GGCTGGACATCGGCCCGCTCCCCACAATGAAATAAAGTTATTTTCTCATTC 291

|||||||||||||||||||||||||||||||||||||||||||||||||||

Sbjct 558 GGCTGGACATCGGCCCGCTCCCCACAATGAAATAAAGTTATTTTCTCATTC 608

1. Ribosomal protein S12

Query 1 ATGGCCGAGGAAGGCATTGCTGCTGGAGGTGTAATGGACGTTAATACTGCTTTACAAGAG 60

||||||||||||||||||||||||||||||||||||||||||||||||||||||||||||

Sbjct 83 ATGGCCGAGGAAGGCATTGCTGCTGGAGGTGTAATGGACGTTAATACTGCTTTACAAGAG 142

Query 61 GTTCTGAAGACTGCCCTCATCCACGATGGCCTAGCACGTGGAATTCGCGAAGCTGCCAAA 120

||||||||||||||||||||||||||||||||||||||||||||||||||||||||||||

Sbjct 143 GTTCTGAAGACTGCCCTCATCCACGATGGCCTAGCACGTGGAATTCGCGAAGCTGCCAAA 202

Query 121 GCCTTAGACAAGCGCCAAGCCCATCTTTGTGTGCTTGCATCCAACTGTGATGAGCCTATG 180

||||||||||||||||||||||||||||||||||||||||||||||||||||||||||||

Sbjct 203 GCCTTAGACAAGCGCCAAGCCCATCTTTGTGTGCTTGCATCCAACTGTGATGAGCCTATG 262

Query 181 TATGTCAAGTTGGTGGAGGCCCTTTGTGCTGAACACCAAATCAACCTAATTAAGGTTGAT 240

||||||||||||||||||||||||||||||||||||||||||||||||||||||||||||

Sbjct 263 TATGTCAAGTTGGTGGAGGCCCTTTGTGCTGAACACCAAATCAACCTAATTAAGGTTGAT 322

Query 241 GACAACAAGAAACTAGGAGAATGGGTAGGCCTTTGTAAAATTGACAGAGAGGGGAAACCC 300

||||||||||||||||||||||||||||||||||||||||||||||||||||||||||||

Sbjct 323 GACAACAAGAAACTAGGAGAATGGGTAGGCCTTTGTAAAATTGACAGAGAGGGGAAACCC 382

Query 301 CGTAAAGTGGTTGGTTGCAGTTGTGTAGTAGTTAAGGACTATGGCAAGGAGTCTCAG-CC 359

||||||||||||||||||||||||||||||||||||||||||||||||||||||||| ||

Sbjct 383 CGTAAAGTGGTTGGTTGCAGTTGTGTAGTAGTTAAGGACTATGGCAAGGAGTCTCAGGCC 442

Query 360 AAGGATGTCATTGAAGAGTATTTCAA-TGCA-GAA-TGA-GAAATAAATCTTTGGCTC 413

|||||||||||||||||||||||||| |||| ||| ||| ||||||||||||||||||

Sbjct 443 AAGGATGTCATTGAAGAGTATTTCAAATGCAAGAAATGAAGAAATAAATCTTTGGCTC 500

1. Ribosomal protein S12

Query 1 ATGGCCGAGGAAGGCATTGCTGCTGGAGGTGTAATGGACGTTAATACTGCTTTACAAGAG 60

||||||||||||||||||||||||||||||||||||||||||||||||||||||||||||

Sbjct 83 ATGGCCGAGGAAGGCATTGCTGCTGGAGGTGTAATGGACGTTAATACTGCTTTACAAGAG 142

Query 61 GTTCTGAAGACTGCCCTCATCCACGATGGCCTAGCACGTGGAATTCGCGAAGCTGCCAAA 120

||||||||||||||||||||||||||||||||||||||||||||||||||||||||||||

Sbjct 143 GTTCTGAAGACTGCCCTCATCCACGATGGCCTAGCACGTGGAATTCGCGAAGCTGCCAAA 202

Query 121 GCCTTAGACAAGCGCCAAGCCCATCTTTGTGTGCTTGCATCCAACTGTGATGAGCCTATG 180

||||||||||||||||||||||||||||||||||||||||||||||||||||||||||||

Sbjct 203 GCCTTAGACAAGCGCCAAGCCCATCTTTGTGTGCTTGCATCCAACTGTGATGAGCCTATG 262

Query 181 TATGTCAAGTTGGTGGAGGCCCTTTGTGCTGAACACCGAATCAACCTAATTAAGATTGAT 240

||||||||||||||||||||||||||||||||||||| |||||||||||||||| |||||

Sbjct 263 TATGTCAAGTTGGTGGAGGCCCTTTGTGCTGAACACCAAATCAACCTAATTAAGGTTGAT 322

Query 241 GACAACAAGAAACTAGGAGAATGGGTAGGCCTTTGTAAAATTGACAGAGAGGGGAAACCC 300

||||||||||||||||||||||||||||||||||||||||||||||||||||||||||||

Sbjct 323 GACAACAAGAAACTAGGAGAATGGGTAGGCCTTTGTAAAATTGACAGAGAGGGGAAACCC 382

Query 301 CGTAAAGTGGTTGGTTGCAGTTGTGTAGTAGTTAAGGACTATGGCAAGGAGTCTCAGGCC 360

||||||||||||||||||||||||||||||||||||||||||||||||||||||||||||

Sbjct 383 CGTAAAGTGGTTGGTTGCAGTTGTGTAGTAGTTAAGGACTATGGCAAGGAGTCTCAGGCC 442

Query 361 AAGGATGTCATTGAAGAGTATTTCAAATGCAAGAAATGAAGAAATAAATCTTTGGCTCAC 420

||||||||||||||||||||||||||||||||||||||||||||||||||||||||||||

Sbjct 443 AAGGATGTCATTGAAGAGTATTTCAAATGCAAGAAATGAAGAAATAAATCTTTGGCTCAC 502

Query 421 A 421

|

Sbjct 503 A 503

1. Ribosomal protein S12

Query 1 ATGGCCGAGGAAGGCATTGCTGCTGGAGGTGTAATGGACGTTAATACTGCTTTACAAGAG 60

||||||||||||||||||||||||||||||||||||||||||||||||||||||||||||

Sbjct 83 ATGGCCGAGGAAGGCATTGCTGCTGGAGGTGTAATGGACGTTAATACTGCTTTACAAGAG 142

Query 61 GTTCTGAAGACTGCCCTCATCCACGATGGCCTAGCACGTGGAATTCGCGAAGCTGCCAAA 120

||||||||||||||||||||||||||||||||||||||||||||||||||||||||||||

Sbjct 143 GTTCTGAAGACTGCCCTCATCCACGATGGCCTAGCACGTGGAATTCGCGAAGCTGCCAAA 202

Query 121 GCCTTAGACAAGCGCCAAGCCCATCTTTGTGTGCTTGCATCCAACTGTGATGAGCCTATG 180

||||||||||||||||||||||||||||||||||||||||||||||||||||||||||||

Sbjct 203 GCCTTAGACAAGCGCCAAGCCCATCTTTGTGTGCTTGCATCCAACTGTGATGAGCCTATG 262

Query 181 TATGTCAAGTTGGTGGAGGCCCTTTGTGCTGAACACCAAATCAACCTAATTAAGGTTGAT 240

||||||||||||||||||||||||||||||||||||||||||||||||||||||||||||

Sbjct 263 TATGTCAAGTTGGTGGAGGCCCTTTGTGCTGAACACCAAATCAACCTAATTAAGGTTGAT 322

Query 241 GACAACAAGAAACTAGGAGAATGGGTAGGCCTTTGTAAAATTGACAGAGAGGGGAAACCC 300

||||||||||||||||||||||||||||||||||||||||||||||||||||||||||||

Sbjct 323 GACAACAAGAAACTAGGAGAATGGGTAGGCCTTTGTAAAATTGACAGAGAGGGGAAACCC 382

Query 301 CGTAAAGTGGTTGGTTGCAGTTGTGTAGTAGTTAAGGACTATGGCAAGGAGTCTCAGGCC 360

||||||||||||||||||||||||||||||||||||||||||||||||||||||||||||

Sbjct 383 CGTAAAGTGGTTGGTTGCAGTTGTGTAGTAGTTAAGGACTATGGCAAGGAGTCTCAGGCC 442

Query 361 AAGGATGTCATTGAAGAGTATTTCAAATGCAAGAAATGAAGAAATAAATCTTTGGCTC 418

||||||||||||||||||||||||||||||||||||||||||||||||||||||||||

Sbjct 443 AAGGATGTCATTGAAGAGTATTTCAAATGCAAGAAATGAAGAAATAAATCTTTGGCTC 500

1. Ribosomal protein S12

Query 1 ATGGCCGAGGAAGGCATTGCTGCTGGAGGTGTAATGGACGTTAATACTGCTTTACAAGAG 60

||||||||||||||||||||||||||||||||||||||||||||||||||||||||||||

Sbjct 83 ATGGCCGAGGAAGGCATTGCTGCTGGAGGTGTAATGGACGTTAATACTGCTTTACAAGAG 142

Query 61 GTTCTGAAGACTGCCCTCATCCACGATGGCCTAGCACGTGGAATTCGCGAAGCTGCCAAA 120

||||||||||||||||||||||||||||||||||||||||||||||||||||||||||||

Sbjct 143 GTTCTGAAGACTGCCCTCATCCACGATGGCCTAGCACGTGGAATTCGCGAAGCTGCCAAA 202

Query 121 GCCTTAGACAAGCGCCAAGCCCATCTTTGTGTGCTTGCATCCAACTGTGATGAGCCTATG 180

||||||||||||||||||||||||||||||||||||||||||||||||||||||||||||

Sbjct 203 GCCTTAGACAAGCGCCAAGCCCATCTTTGTGTGCTTGCATCCAACTGTGATGAGCCTATG 262

Query 181 TATGTCAAGTTGGTGGAGGCCCTTTGTGCTGAACACCAAATCAACCTAATTAAGGTTGAT 240

||||||||||||||||||||||||||||||||||||||||||||||||||||||||||||

Sbjct 263 TATGTCAAGTTGGTGGAGGCCCTTTGTGCTGAACACCAAATCAACCTAATTAAGGTTGAT 322

Query 241 GACAACAAGAAACTAGGAGAATGGGTAGGCCTTTGTAAAATTGACAGAGAGGGGAAACCC 300

||||||||||||||||||||||||||||||||||||||||||||||||||||||||||||

Sbjct 323 GACAACAAGAAACTAGGAGAATGGGTAGGCCTTTGTAAAATTGACAGAGAGGGGAAACCC 382

Query 301 CGTAAAGTGGTTGGTTGTAGTTGTGTAGTAGTTAAGGACTATGGCAAGGAGTCTCAGGCC 360

||||||||||||||||| ||||||||||||||||||||||||||||||||||||||||||

Sbjct 383 CGTAAAGTGGTTGGTTGCAGTTGTGTAGTAGTTAAGGACTATGGCAAGGAGTCTCAGGCC 442

Query 361 AAGGATGTCATTGAAGAGTATTTCAAATGCAAGAAATGAAGAAATAAATCTTTGGCTCAC 420

||||||||||||||||||||||||||||||||||||||||||||||||||||||||||||

Sbjct 443 AAGGATGTCATTGAAGAGTATTTCAAATGCAAGAAATGAAGAAATAAATCTTTGGCTCAC 502

Query 421 A 421

|

Sbjct 503 A 503

1. Ribosomal protein S28

Query 1 ATGGACACCAGCCGTGTGCAGCCTATCAAGCTGGCCAGGGTCACCAAGGTCCTGGGCAGG 60

||||||||||||||||||||||||||||||||||||||||||||||||||||||||||||

Sbjct 32 ATGGACACCAGCCGTGTGCAGCCTATCAAGCTGGCCAGGGTCACCAAGGTCCTGGGCAGG 91

Query 61 ACCGGTTCTCAGGGACAGTGCACGCAGGTGCGCGTGGAATTCATGGACGACACGAGCCGA 120

||||||||||||||||||||||||||||||||||||||||||||||||||||||||||||

Sbjct 92 ACCGGTTCTCAGGGACAGTGCACGCAGGTGCGCGTGGAATTCATGGACGACACGAGCCGA 151

Query 121 TCCATCATCCGCAATGTAAAAGGCCCCGTGCGCGAGGGCGACGTGCTCACCCTTTTGGAG 180

||||||||||||||||||||||||||||||||||||||||||||||||||||||||||||

Sbjct 152 TCCATCATCCGCAATGTAAAAGGCCCCGTGCGCGAGGGCGACGTGCTCACCCTTTTGGAG 211

Query 181 TCAGAGCGAGAAGCCCGGAGGTTGCGCTGAGCTTGGCTGCTCGCTGGGTCTTGGATGTCG 240

||||||||||||||||||||||||||||||||||||||||||||||||||||||||||||

Sbjct 212 TCAGAGCGAGAAGCCCGGAGGTTGCGCTGAGCTTGGCTGCTCGCTGGGTCTTGGATGTCG 271

Query 241 GGTTCGACCACTTGGCCGATGGGAATGGTCTGTCACAATCTGCTCCTTTTTTTTGTCCGC 300

||||||||||||||||||||||||||||||||||||| ||||||||||||||||||||||

Sbjct 272 GGTTCGACCACTTGGCCGATGGGAATGGTCTGTCACAGTCTGCTCCTTTTTTTTGTCCGC 331

Query 301 CACACGTAACTGAGATGCTCCTTTAAATAAAGCGTTTGTGTTTCAAG 347

|||||||||||||||||||||||||||||||||||||||||||||||

Sbjct 332 CACACGTAACTGAGATGCTCCTTTAAATAAAGCGTTTGTGTTTCAAG 378

1. Ribosomal protein S28

Query 1 ATGGACACCAGCCGTGTGCAGCCTATCAAGCTGGCCAGGGTCACCAAGGTCCTGGGCAGG 60

||||||||||||||||||||||||||||||||||||||||||||||||||||||||||||

Sbjct 32 ATGGACACCAGCCGTGTGCAGCCTATCAAGCTGGCCAGGGTCACCAAGGTCCTGGGCAGG 91

Query 61 ACCGGTTCTCAGGGACAGTGCACGCAGGTGCGCGTGGAATTCATGGACGACACGAGCCGA 120

||||||||||||||||||||||||||||||||||||||||||||||||||||||||||||

Sbjct 92 ACCGGTTCTCAGGGACAGTGCACGCAGGTGCGCGTGGAATTCATGGACGACACGAGCCGA 151

Query 121 TCCATCATCCGCAATGTAAAAGGCCCCGTGCGCGAGGGCGACGTGCTCACCCTTTTGGAG 180

||||||||||||||||||||||||||||||||||||||||||||||||||||||||||||

Sbjct 152 TCCATCATCCGCAATGTAAAAGGCCCCGTGCGCGAGGGCGACGTGCTCACCCTTTTGGAG 211

Query 181 TCAGAGCGAGAAGCCCGGAGGTTGCGCTGAGCTTGGCTGCTCGCTGGGTCTTGGATGTCG 240

||||||||||||||||||||||||||||||||||||||||||||||||||||||||||||

Sbjct 212 TCAGAGCGAGAAGCCCGGAGGTTGCGCTGAGCTTGGCTGCTCGCTGGGTCTTGGATGTCG 271

Query 241 GGTTCGACCACTTGGCCGATGGGAATGGTCTGTCACAATCTGCTCCTTTTTTTTGTCCGC 300

||||||||||||||||||||||||||||||||||||| ||||||||||||||||||||||

Sbjct 272 GGTTCGACCACTTGGCCGATGGGAATGGTCTGTCACAGTCTGCTCCTTTTTTTTGTCCGC 331

Query 301 CACACGTAACTGAGATGCTCCTTTAAATAAAGCGTTTGTGTTTCAAG 347

|||||||||||||||||||||||||||||||||||||||||||||||

Sbjct 332 CACACGTAACTGAGATGCTCCTTTAAATAAAGCGTTTGTGTTTCAAG 378

1. Ribosomal protein S28

Query 1 ATGGACACCAGCCGTGTGCAGCCTATCAAGCTGGCCAGGGTCACCAAGGTCCTGGGCAGG 60

||||||||||||||||||||||||||||||||||||||||||||||||||||||||||||

Sbjct 32 ATGGACACCAGCCGTGTGCAGCCTATCAAGCTGGCCAGGGTCACCAAGGTCCTGGGCAGG 91

Query 61 ACCGGTTCTCAGGGACAGTGCACGCAGGTGCGCGTGGAATTCATGGACGACACGAGCCGA 120

||||||||||||||||||||||||||||||||||||||||||||||||||||||||||||

Sbjct 92 ACCGGTTCTCAGGGACAGTGCACGCAGGTGCGCGTGGAATTCATGGACGACACGAGCCGA 151

Query 121 TCCATCATCCGCAATGTAAAAGGCCCCGTGCGCGAGGGCGACGTGCTCACCCTTTTGGAG 180

||||||||||||||||||||||||||||||||||||||||||||||||||||||||||||

Sbjct 152 TCCATCATCCGCAATGTAAAAGGCCCCGTGCGCGAGGGCGACGTGCTCACCCTTTTGGAG 211

Query 181 TCAGAGCGAGAAGCCCGGAGGTTGCGCTGAGCTTGGCTGCTCGCTGGGTCTTGGATGTCG 240

||||||||||||||||||||||||||||||||||||||||||||||||||||||||||||

Sbjct 212 TCAGAGCGAGAAGCCCGGAGGTTGCGCTGAGCTTGGCTGCTCGCTGGGTCTTGGATGTCG 271

Query 241 GGTTCGACCACTTGGCCGATGGGAATGGTCTGTCACAATCTGCTCCTTTTTTTTGTCCGC 300

||||||||||||||||||||||||||||||||||||| ||||||||||||||||||||||

Sbjct 272 GGTTCGACCACTTGGCCGATGGGAATGGTCTGTCACAGTCTGCTCCTTTTTTTTGTCCGC 331

Query 301 CACACGTAACTGAGATGCTCCTTTAAATAAAGCGTTTGTGTTTCAAG 347

|||||||||||||||||||||||||||||||||||||||||||||||

Sbjct 332 CACACGTAACTGAGATGCTCCTTTAAATAAAGCGTTTGTGTTTCAAG 378

1. Ribosomal protein L22

Query 1 ATGGCTCCTGTGAAAAAGCTTGTGGTGAAGGGGGGCAAAAAAAAGAAGCAAGTTCTGAAG 60

||||||||||||||||||||||||||||||||||||||||||||||||||||||||||||

Sbjct 47 ATGGCTCCTGTGAAAAAGCTTGTGGTGAAGGGGGGCAAAAAAAAGAAGCAAGTTCTGAAG 106

Query 61 TTCACTCTTGATTGCACCCACCCTGTAGAAGATGGAATCATGGATGCTGCCAATTTCGAG 120

|||||||||||||||||||||||||||||||||||||||||||||||||||||||| |||

Sbjct 107 TTCACTCTTGATTGCACCCACCCTGTAGAAGATGGAATCATGGATGCTGCCAATTTTGAG 166

Query 121 CAGTTTTTGCAAGAAAGGATCAAAGTGAACGGAAAAGCTGGGAACCTTGGTGGAGGGGTG 180

||||||||||||||||||||||||||||||||||||||||||||||||||||||||||||

Sbjct 167 CAGTTTTTGCAAGAAAGGATCAAAGTGAACGGAAAAGCTGGGAACCTTGGTGGAGGGGTG 226

Query 181 GTGACCATCGAAAGGAGCAAGAGCAAGATCACCGCGACATCCGAGGTGCCTTTCTCCAAA 240

|||||||||||||||||||||||||||||||||| |||||||||||||||||||||||||

Sbjct 227 GTGACCATCGAAAGGAGCAAGAGCAAGATCACCGTGACATCCGAGGTGCCTTTCTCCAAA 286

Query 241 AGGTATTTGAAATATCTCACCAAAAAATATTTGAAGAAGAATAATCTACGTGACTGGTTG 300

||||||||||||||||||||||||||||||||||||||||||||||||||||||||||||

Sbjct 287 AGGTATTTGAAATATCTCACCAAAAAATATTTGAAGAAGAATAATCTACGTGACTGGTTG 346

Query 301 CGCGTAGTTGCTAACAGCAAAGAGAGTTACGAATTACGTTACTTCCAGATTAACCAGGAC 360

||||||||||||||||||||||||||||||||||||||||||||||||||||||||||||

Sbjct 347 CGCGTAGTTGCTAACAGCAAAGAGAGTTACGAATTACGTTACTTCCAGATTAACCAGGAC 406

Query 361 GAAGAAGAGGAGGAAGACGAGGATTAAATTTCATTTATCTGGAAAATTTTGTATGAGTTC 420

||||||||||||||||||||||||||||||||||||||||||||||||||||||||||||

Sbjct 407 GAAGAAGAGGAGGAAGACGAGGATTAAATTTCATTTATCTGGAAAATTTTGTATGAGTTC 466

Query 421 TTGAATAAAACTTGG 435

|||||||||||||||

Sbjct 467 TTGAATAAAACTTGG 481

1. Ribosomal protein, large, P1

Query 1 ATGGCCTCTGTCTCCGAGCTCGCCTGCATCTACTCGGCCCTCATTCTGCACGACGATGAG 60

||||||||||||||||||||||||||||||||||||||||||||||||||||||||||||

Sbjct 130 ATGGCCTCTGTCTCCGAGCTCGCCTGCATCTACTCGGCCCTCATTCTGCACGACGATGAG 189

Query 61 GTGACAGTCACGGAGGATAAGATCAATGCCCTCATTAAAGCAGCCGGTGTAAATGTTGAG 120

||||||||||||||||||||||||||||||||||||||||||||||||||||||||||||

Sbjct 190 GTGACAGTCACGGAGGATAAGATCAATGCCCTCATTAAAGCAGCCGGTGTAAATGTTGAG 249

Query 121 CCTTTTTGGCCTGGCTTGTTTGCAAAGGCCCTGGCCAACGTCAACATTGGGAGCCTCATC 180

||||||||||||||||||||||||||||||||||||||||||||||||||||||||||||

Sbjct 250 CCTTTTTGGCCTGGCTTGTTTGCAAAGGCCCTGGCCAACGTCAACATTGGGAGCCTCATC 309

Query 181 TGCAATGTAGGGGCCGGTGGACCTGCTCCAGCAGCTGGTGCTGCACCAGCAGGAGGTCCT 240

||||||||||||||||||||||||||||||||||||||||||||||||||||||||||||

Sbjct 310 TGCAATGTAGGGGCCGGTGGACCTGCTCCAGCAGCTGGTGCTGCACCAGCAGGAGGTCCT 369

Query 241 GCCCCCTCCACTGCTGCTGCTCCAGCTGAGGAGAAGAAAGTGGAAGCAAAGAAAGAAGAA 300

||||||||||||||||||||||||||||||||||||||||||||||||||||||||||||

Sbjct 370 GCCCCCTCCACTGCTGCTGCTCCAGCTGAGGAGAAGAAAGTGGAAGCAAAGAAAGAAGAA 429

Query 301 TCCGAGGAGTCTGATGATGACATGGGCTTTGGTCTTTTTGACTAAACCTCTTTTATAACA 360

||||||||||||||||||||||||||||||||||||||||||||||||||||||||||||

Sbjct 430 TCCGAGGAGTCTGATGATGACATGGGCTTTGGTCTTTTTGACTAAACCTCTTTTATAACA 489

Query 361 TGTTCAATAAAAAGCTGAACTTT 383

|||||||||||||||||||||||

Sbjct 490 TGTTCAATAAAAAGCTGAACTTT 512

1. Chromosome 5 genomic contig

Query 1 ATGGGAACACAGTTGTCGGGTTGACTCACTGAGTAACAGAAAGAGAAAAGCATCTCAGAA 60

||||||||||||||||||||||||||||||||||||||||||||||||||||||||||||

Sbjct 45815642 ATGGGAACACAGTTGTCGGGTTGACTCACTGAGTAACAGAAAGAGAAAAGCATCTCAGAA 701

Query 61 GCTTATTGGGTACAAAGCGACCACCTTGAGATCACATAAGCGCTACAGACAGATGTCAAA 120

||||||||||||||||||||||||||||||||||||||||||||||||||||||||||||

Sbjct 45815702 GCTTATTGGGTACAAAGCGACCACCTTGAGATCACATAAGCGCTACAGACAGATGTCAAA 761

Query 121 TATCCAAACACTTAAGGTCAGAAAGAGTCATCAACATGTGCCTATGTTTTCTTCTCCCCA 180

||||||||||||||||||||||||||||||||||||||||||||||||||||||||||||

Sbjct 45815762 TATCCAAACACTTAAGGTCAGAAAGAGTCATCAACATGTGCCTATGTTTTCTTCTCCCCA 821

Query 181 GTTTTATCTAGAAAGGTGAGAACATATTTTCAAGCCTGCTCATATCTGTATACTTAGCCT 240

||||||||||||||||||||||||||||||||||||||||||||||||||||||||||||

Sbjct 45815822 GTTTTATCTAGAAAGGTGAGAACATATTTTCAAGCCTGCTCATATCTGTATACTTAGCCT 881

Query 241 CTTCCTGCAATACATAAATGCCCACCCACCTCTTCTCACATCTATTTCTAAGGATATTTA 300

||||||||||||||||||||||||||||||||||||||||||||||||||||||||||||

Sbjct 45815882 CTTCCTGCAATACATAAATGCCCACCCACCTCTTCTCACATCTATTTCTAAGGATATTTA 941

Query 301 TTTCTAAGGATAAAGATGGCACATGTCTATGTAGAACAGAGAAGAAGCATCTGAATCGGA 360

||||||||||||||||||||||||||||||||||||||||||||||||||||||||||||

Sbjct 45815942 TTTCTAAGGATAAAGATGGCACATGTCTATGTAGAACAGAGAAGAAGCATCTGAATCGGA 001

Query 361 GCTGATGCTCTTGGTCATTTTTCTCTAACTAAAGCTCAAAATGAAATTTTAGATCTAGTG 420

||||||||||||||||||||||||||||||||||||||||||||||||||||||||||||

Sbjct 45816002 GCTGATGCTCTTGGTCATTTTTCTCTAACTAAAGCTCAAAATGAAATTTTAGATCTAGTG 061

Query 421 GCTGCTCAGGGACGCACCTGGAGCTACACTTTCAGTAGTGGAGACAGCCGGAGCAGAGGA 480

||||||||||||||||||||||||||||||||||||||||||||||||||||||||||||

Sbjct 45816062 GCTGCTCAGGGACGCACCTGGAGCTACACTTTCAGTAGTGGAGACAGCCGGAGCAGAGGA 121

Query 481 TGGTGCTGGCAGCGCAGGCATCATGACAATGGTAGCTTGGGACACAGACTCTACAGGGGG 540

||||||||||||||||||||||||||||||||||||||||||||||||||||||||||||

Sbjct 45816122 TGGTGCTGGCAGCGCAGGCATCATGACAATGGTAGCTTGGGACACAGACTCTACAGGGGG 181

Query 541 TGGCACAAGTTTAACTGGAGGCTCACTGGCAACAGTAGTGAAGGAAGCAAGAGGTGTGGT 600

||||||||||||||||||||||||||||||||||||||||||||||||||||||||||||

Sbjct 45816182 TGGCACAAGTTTAACTGGAGGCTCACTGGCAACAGTAGTGAAGGAAGCAAGAGGTGTGGT 241

Query 601 GAACTGTGTAGGACCAGCTTCTAAAAGCCGGGAAAGAGTGGGAGCACCTAACATATAAAG 660

||||||||||||||||||||||||||||||||||||||||||||||||||||||||||||

Sbjct 45816242 GAACTGTGTAGGACCAGCTTCTAAAAGCCGGGAAAGAGTGGGAGCACCTAACATATAAAG 301

Query 661 AGGTACACAAAATGAAGTAAGAAGAAAGCACATAAATAAGAAGCAATAGCTACTTGAAGA 720

||||||||||||||||||||||||||||||||||||||||||||||||||||||||||||

Sbjct 45816302 AGGTACACAAAATGAAGTAAGAAGAAAGCACATAAATAAGAAGCAATAGCTACTTGAAGA 361

Query 721 CTCAACTAAGAAAATTCTTATGGTCTCAAAGGATGAGAGCAACTCCTGCATTGAATAAGT 780

||||||||||||||||||||||||||||||||||||||||||||||||||||||||||||

Sbjct 45816362 CTCAACTAAGAAAATTCTTATGGTCTCAAAGGATGAGAGCAACTCCTGCATTGAATAAGT 421

Query 781 TCATCATCTATATCCTTACTCTAACCTTACTTAAGATCTTCCTCCAAGTTAGGAATGAAC 840

||||||||||||||||||||||||||||||||||||||||||||||||||||||||||||

Sbjct 45816422 TCATCATCTATATCCTTACTCTAACCTTACTTAAGATCTTCCTCCAAGTTAGGAATGAAC 481

Query 841 CAAAGCACTAGAACACACAACATATATAAAAAATGGGTGGTAAGGCTAGGTGCAGTGGCT 900

||||||||||||||||||||||||||||||||||||||||||||||||||||||||||||

Sbjct 45816482 CAAAGCACTAGAACACACAACATATATAAAAAATGGGTGGTAAGGCTAGGTGCAGTGGCT 541

Query 901 CACGCCTGTAATGCTAGCACTTTGGGAGGCCGAGGCAG-TGGGTCACTTGAGGT 953

|||||||||||||||||||||||||||||||||||||| |||||||||||||||

Sbjct 45816542 CACGCCTGTAATGCTAGCACTTTGGGAGGCCGAGGCAGGTGGGTCACTTGAGGT 45816595

1. Chromosome 2 genomic contig

Query 1 ATGTGAGAGGACCTGAGTTTACATATCAGGCTGATCCTTGCCATAGACATGGTCTATAAC 60

||||||||||||||||||||||||||||||||||||||||||||||||||||||||||||

Sbjct 51393703 ATGTGAGAGGACCTGAGTTTACATATCAGGCTGATCCTTGCCATAGACATGGTCTATAAC 644

Query 61 AATCAACAAAACAATCAACAAAAACGGTAACAAAAAACAGCAAGTCCTAGAGAAGGGGAA 120

||||||||||||||||||||||||||||||||||||||||||||||||||||||||||||

Sbjct 51393643 AATCAACAAAACAATCAACAAAAACGGTAACAAAAAACAGCAAGTCCTAGAGAAGGGGAA 584

Query 121 GAATTTTTTTTCCAAAGTTGCCACATTATTAGATTCAAATGTCCAGTTTTCAATTAAAAA 180

||||||||||||||||||||||||||||||||||||||||||||||||||||||||||||

Sbjct 51393583 GAATTTTTTTTCCAAAGTTGCCACATTATTAGATTCAAATGTCCAGTTTTCAATTAAAAA 524

Query 181 TCGCAAGGAATACAAAGAAACAGGAAAGTATTGCCCATGCAAGC 224

||||||||||||||||||||||||||||||||||||||||||||

Sbjct 51393523 TCGCAAGGAATACAAAGAAACAGGAAAGTATTGCCCATGCAAGC 51393480

1. Short DNA

Query 5 ATGGGCTCCTGAGCCCCCTGCCCCCAGAGCAATAAAGTCAGCTGGCTTTCTCA 53

|||||||||||||||||||||||||||||||||||||||||||||||||

Sbjct 643 GCTCCTGAGCCCCCTGCCCCCAGAGCAATAAAGTCAGCTGGCTTTCTCA 691

1. Short DNA

Query 5 ATGGGCTCCTGAGCCCCCTGCCCCCAGAGCAATAAAGTCAGCTGGCTTTC 50

||||||||||||||||||||||||||||||||||||||||||||||

Sbjct 643 GCTCCTGAGCCCCCTGCCCCCAGAGCAATAAAGTCAGCTGGCTTTC 688

1. Short DNA

Query 5 ATGGGCTCCTGAGCCCCCTGCCCCCAGAGCAATAAAGTCAGCTGGCTTTC 50

||||||||||||||||||||||||||||||||||||||||||||||

Sbjct 643 GCTCCTGAGCCCCCTGCCCCCAGAGCAATAAAGTCAGCTGGCTTTC 688

1. Short DNA

Query 5 ATGGGCTCCTGAGCCCCCTGCCCCCAGAGCAATAAAGTCAGCTGGCTTTC 50

||||||||||||||||||||||||||||||||||||||||||||||

Sbjct 643 GCTCCTGAGCCCCCTGCCCCCAGAGCAATAAAGTCAGCTGGCTTTC 688

1. Short DNA

Query 1 ATGTGTGTTGATACTGTTGCACGTGTGTTTTTCTATTAAAAGACTCATCCG 51

|||||||||||||||||||||||||||||||||||||||||||||||||||

Sbjct 1108 ATGTGTGTTGATACTGTTGCACGTGTGTTTTTCTATTAAAAGACTCATCCG 1158

1. Short DNA

ATGGCCTCCATGACTTTGCAAAAAAAAAAAAAAAAAAAAAAAAAAAAAA

1. Short DNA

ATGACTTTTTAAAAAAAAAAAAAAAGCAAAAAAAAAAAAAAAAAAAAAAAAAAAAA

1. Short DNA

ATGTTCAGTTTGCAAAAAAAAAAAAAAAAAAAAAAAAAAAAAA

1. Short DNA

ATGTTCAGTTTGCAAAAAAAAAAAAAAAAAAAAAAAAAAAAAA
