# Supplementary material for: In-Frame cDNA Library Combined with Protein Complementation Assay Identifies ARL11-Binding Partners
Source: PLoS One. 2012 Dec 18;7(12):e52290. doi: 10.1371/journal.pone.0052290 (PMC3525598; doi:10.1371/journal.pone.0052290)
Supplement: Table S4 — Clones identified as putative ARL11 binders. (DOC) [file pone.0052290.s004.doc]

**Table S4. C**lones identified as putative ARL11 binders

| **Ref sequence number** | **Gene name** | **Number of clones** |
| --- | --- | --- |
| NM_001878.3 | Retinoic acid binding protein 2 (CRABP2) | 3 |
| NM_002629.2 | Phosphoglycerate mutase 1(PGAM1) | 1 |
| NM_144582.2 | Testis expressed 261 (TEX261) | 1 |
| XR_111191.2 | Hypothetical LOC100507645, transcript variant 2 | 1 |
| NM_001015.3 | Ribosomal protein S11 (RPS11) | 1 |
| [NM_001016.3](http://www.ncbi.nlm.nih.gov/nucleotide/71164877?report=genbank&log$=nuclalign&blast_rank=1&RID=7H5399DC016) | Ribosomal protein S12 (RPS12) | 4 |
| NM_001031.4 | Ribosomal protein S28 (RPS28) | 3 |
| [NM_000983.3](http://www.ncbi.nlm.nih.gov/nucleotide/48255919?report=genbank&log$=nuclalign&blast_rank=1&RID=7H5RDWR6016) | Ribosomal protein L22 (RPL22) | 1 |
| NM_001003.2 | Ribosomal protein, large, P1 (RPLP1) | 1 |
| NT_034772.6 | Chromosome 5 genomic contig | 1 |
| NT_022184.15 | Chromosome 2 genomic contig | 1 |
|  | Short DNA | 9 |
